# Valorization systems based on electrocatalytic nitrate/nitrite conversion for energy supply and valuable product synthesis

**DOI:** 10.1039/d4sc05936k

**Published:** 2024-11-29

**Authors:** Yi Feng, Jin-Tao Ren, Ming-Lei Sun, Zhong-Yong Yuan

**Affiliations:** a School of Materials Science and Engineering, Smart Sensing Interdisciplinary Science Center, Nankai University Tianjin 300350 China zyyuan@nankai.edu.cn

## Abstract

The excessive accumulation of nitrate/nitrite (NO_*x*_^−^) in surface and groundwater has severely disrupted the global nitrogen cycle and jeopardized public health. The electrochemical conversion of NO_*x*_^−^ to ammonia (NH_3_) not only holds promise for ecofriendly NO_*x*_^−^ removal, but also provides a green alternative to the energy-intensive Haber–Bosch process for NH_3_ production. Recently, in addition to the electrocatalyst design explosion in this field, many innovative valorization systems based on NO_*x*_^−^-to-NH_3_ conversion have been developed for generating energy and expanding the range of value-added products. Collective knowledge of advanced conversion systems is indispensable for restoring the global nitrogen cycle and promoting a N-based economy. Herein, a timely and comprehensive review is provided on the important progress of valorization systems based on NO_*x*_^−^ conversion, including waste treatment systems, novel electrolytic systems, and energy conversion and storage systems. Some mechanism explorations, device designs, key electrode developments and feasibility analyses are involved to gain deeper understanding of various systems and facilitate implementing these cleaning systems in industry. Finally, challenges and future prospects are outlined in the NO_*x*_^−^ conversion field with an aim to promote large-scale electrocatalytic system development and prosperous N-based electrochemistry.

## Abstract 2

Recent progress of valorization systems based on NO_*x*_^−^ conversion, including waste treatment systems, novel electrolytic systems, and energy conversion and storage systems, is reviewed, and the challenges and future prospects are outlined.

## Graphical abstract

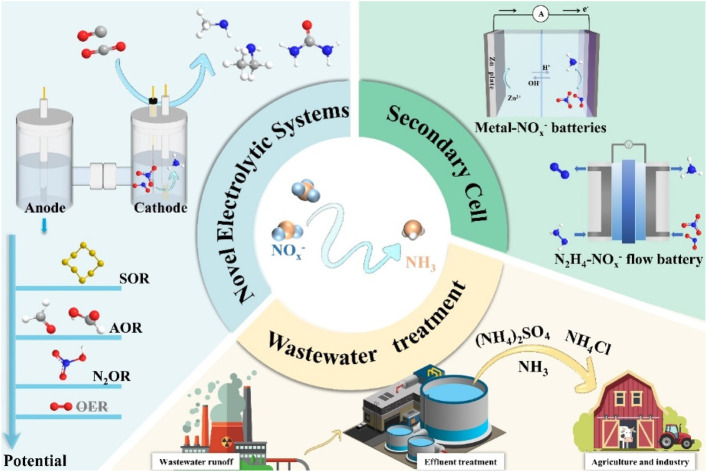

## 1. Introduction

Nitrogen is an indispensable element in the biological processes of organisms, and the global nitrogen cycle plays a crucial role in material exchange within the biosphere.^1–5^ The severe disruption of the global nitrogen cycle is caused by the abusive use of synthetic N-containing fertilizers and chemicals,^6–8^ causing excessive accumulation of N-species such as nitrate/nitrite (NO_*x*_^−^) in surface and groundwater, which may lead to eutrophic water bodies, devastated aquatic ecosystems and jeopardized public health.^9^ For restoring the global nitrogen cycle and alleviating the pollutant effect, an electrochemistry-assisted strategy has surged as a promising approach for the degradation of NO_*x*_^−^ in wastewater,^10^ in contrast with the most widely employed conventional biotechnology method requiring an adequate organic carbon source,^11^ which stands at the midpoint of pursuing sustainable NO_*x*_^−^ removal and carbon neutrality.^12–14^ Compared with the direct conversion to harmless and low-value N_2_,^15^ more and more efforts are being devoted to electrocatalytic conversion of NO_*x*_^−^ to NH_3_ due to easier N–H bond formation and more valuable products.

**Fig. 1:**
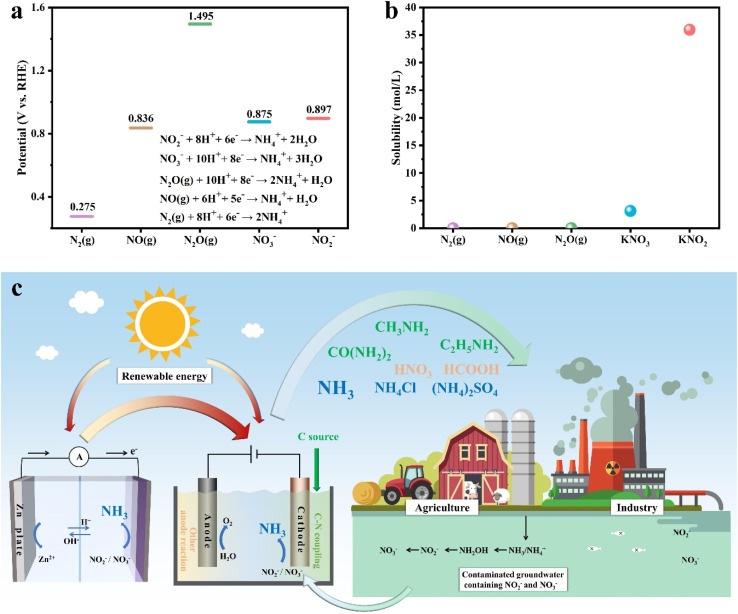
(a) Theoretical potentials of the reduction reactions of N_2_, NO, N_2_O, NO_3_^−^ and NO_2_^−^ to NH_4_^+^ at the same pH (1). (b) The solubility of N_2_, NO, N_2_O, KNO_3_ and KNO_2_ in water at room temperature. (c) The various valorization systems of electrocatalytic nitrate/nitrite conversion for energy storage and conversion, and synthesis of multiple valuable products. Red arrow: the direction of the flow of energy; green arrow: the direction of the flow of matter.

NH_3_ is a versatile chemical raw material accounting for 5% of the chemical market value and has also been acknowledged as an intriguing carbon-free energy carrier containing 17.5 wt% hydrogen (H_2_).^16^ However, industrial synthesis of NH_3_ heavily relies on the Haber–Bosch process which consumes a considerable amount of energy of 5.5 EJ per year and emits about 3.0 t of CO_2_ per metric ton of NH_3_ produced.^17^ Thus, further advancements are necessary to achieve lower temperature ammonia synthesis with a low or zero carbon footprint. So far, several attractive routes for electrocatalytic NH_3_ synthesis under ambient conditions have been proposed, which can be achieved by electrochemical reduction of N-containing species such as nitrogen (N_2_)^18^ and nitrogen oxides (NO, N_2_O).^19^ However, the electrochemical reduction efficiencies of N_2_, NO and N_2_O are limited by the vigorous competition from the H_2_ evolution reaction (HER) due to their ultralow solubility in water (Fig. 1a and b). In contrast, electrocatalytic reduction reactions of NO_*x*_^−^ are much easier to mitigate against the HER competition due to their higher solubility and more positive potentials.^20^

The period since 2020 has witnessed an explosive growth in the literature devoted to designing advanced electrocatalysts with high faradaic efficiency (FE) and NH_3_ yield rate, reflecting a comprehensive and intensive exploration of diverse electrode materials.^21–23^ In recent years, a variety of valorization systems (Fig. 1c) based on electrochemical NO_*x*_^−^-to-NH_3_ conversion have been developed,^24–27^ including sewage treatment systems for NO_*x*_^−^ removal and systems for producing NH_3_-based chemicals, energy storage systems including metal–NO_*x*_^−^ batteries and N_2_H_4_–NO_*x*_^−^ batteries for energy supply and storing intermittent renewable energies, and novel electrolytic systems for production of multiple value-added chemicals. The design of novel electrolytic systems can include the modification and substitution of anode and cathode reactions. According to the cathodic reaction, integrated and tandem reactions based on NO_*x*_^−^ reduction and C species conversion can yield high-value-added chemicals such as urea^28^ and methylamine,^29^ which are generally synthesized through energy- and emission-intensive processes. More intriguingly, the anodic reaction is commonly the oxygen evolution reaction (OER) in an electrolyzer for electrocatalytic NO_3_^−^/NO_2_^−^ reduction to NH_3_, which possesses sluggish kinetics and produces low-value O_2_ ($25 per ton).^30^ Numerous reactions including oxidation of small organic molecules could then be employed to replace the anodic OER for reducing overall energy consumption and obtaining other high-value-added products.^31^

In view of the significance of the collective knowledge on advanced conversion systems for prosperous N-based chemistry and the scarcity of systematic reviews towards NO_*x*_^−^ reduction applications, a timely and comprehensive review is provided on the recent fundamental insights and achievements of valorization systems based on NO_*x*_^−^ conversion, including waste treatment systems, novel electrolytic systems, and energy conversion and storage systems. In this review, the basic knowledge of NO_*x*_^−^ reduction is firstly provided including reaction mechanisms, reaction devices and design principles of catalysts. Then, the obtained technological innovations and existing challenges are elaborated on with regards to the applications of NO_*x*_^−^ reduction by summarizing mechanism explorations, key electrode developments, and feasibility analyses. Finally, challenges and future prospects are outlined in the NO_*x*_^−^ conversion field with an aim to promote large-scale electrocatalytic system development and prosperous N-based electrochemistry.

## 2. Fundamentals of electrocatalytic NO_3_^−^/NO_2_^−^ reduction

### 2.1 Reaction mechanisms

**Fig. 2:**
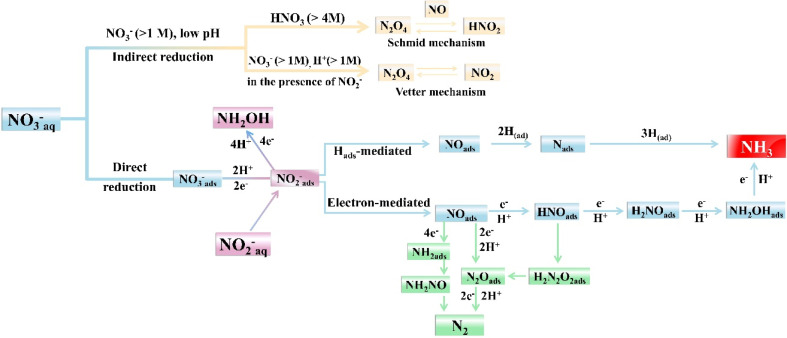
Mechanisms and main processes during electrocatalytic NO_3_^−^/NO_2_^−^ reduction in water.

Recognizing and mastering the mechanisms of the electrocatalytic conversions of NO_3_^−^ and NO_2_^−^ is a prerequisite for the development of self-powered denitrification systems. Electrochemical reduction of NO_3_^−^ is a complex process with multielectron reactions,^32^ which involves various nitrogen-containing intermediates and products ranging from −3 to +5 valence states. N_2_ and NH_3_/NH_4_^+^ are widely recognized as the most thermodynamically stable products,^33^ but the final result can be altered due to several causes, such as cathode materials and the pH value of the solution. The indirect autocatalytic reduction mechanism and the direct electrocatalytic reduction mechanism comprise the currently accepted mechanism of NO_3_^−^ electroreduction.^34^ As depicted in Fig. 2, the indirect autocatalytic reaction is considered as the reduction process without NO_3_^−^ involved in the electron transfer, only existing under the conditions of nitrate concentration exceeding 1 M and low pH value. However, more efforts are put into the mechanism of NO_3_^−^ electroreduction occurring at concentrations lower than 1 M (direct reaction).^35^ The active adsorbed hydrogen atom (H_ads_)-mediated route and the electron-mediated pathway are pursued simultaneously in the direct mechanism (Fig. 2), which leads to the complexity of the electrocatalytic NO_3_^−^ reduction.

The electroreduction of NO_3_^−^ is initiated by the adsorption of NO_3_^−^ ions onto the cathodic electrodes. The adsorbed NO_3_^−^ is transformed into NO_2_^−^ by a tripartite electrochemical–chemical–electrochemical process, which is recognized as the dominant rate-controlling step.^36^ Later, the nitric oxide (NO_ads_) intermediate is derived by NO_2_^−^ conversion. As depicted in Fig. 2, NO_ads_ can be reduced to NH_3_ as the ultimate product and occupy a dominant position in the N_2_ formation pathway.

In addition, the reduction process of NO_3_^−^ can be mediated by H_ads_. NO_2ads_^−^, NO_3_^−^, and NO_ads_ can be reduced by H_ads_.^15^ The predominant final product in this H_ads_-mediated process is NH_3_, which is caused by the fact that the formation of N–N bonds mediated by H_ads_ is kinetically more challenging than the formation of N–H bonds. The specific H_ads_-mediated pathways are described by reactions (1)–(7).

1 H_2_O + e^−^ → H_ads_ + OH^−^

2 NO_3_^−^ + 2H_ads_ → NO_2ads_^−^ + H_2_O

3 NO_2ads_^−^ + H_ads_ → NO_ads_ + OH^−^

4 NO_ads_ + 2H_ads_ → N_ads_ + H_2_O

5 N_ads_ + H_ads_ → NH_ads_

6 NH_ads_ + H_ads_ → NH_2ads_

7 NH_2ads_ + H_ads_ → NH_3ads_

NO_ads_ is also essential for the formation of N_2_. As illustrated in eqn (8)–(11), unstable HN_2_O_2_ can be formed by NO_ads_, sequentially forming N_2_O_ads_ and N_2_ through electron transfer. The generation of N_2_ can also be obtained by the rapid decomposition of NH_2_NO. It is known from eqn (12)–(14) that the stable NH_2ads_ can be formed through the NO_ads_ reduction process, which can react with NO_ads_ to form NH_2_NO.

8 NO_ads_ + NO_ads_ + e^−^ + H^+^ → HN_2_O_2_

9 HN_2_O_2ads_ + e^−^ + H^+^ → N_2_O_ads_ + H_2_O

10 N_2_O + e^−^ → N_2_O^−^

11 N_2_O^−^ + e^−^ + 2H^+^ → N_2_ + H_2_O

12 NO_ads_ + 3H_2_O + 4e^−^ → NH_2ads_ + 4OH^−^

13 NO_ads_ + NH_2ads_ → NH_2_NO_ads_

14 NH_2_NO_ads_ → N_2_ + H_2_O

The electrocatalytic NO_2_^−^ reduction to NH_3_ is roughly identical to the reduction process following the conversion of NO_3_^−^ to NO_2_^−^. In fact, NO_2_^−^ reduction is easier than NO_3_^−^reduction due to less charge transfer involved. In the NO_3_^−^ reduction process on many catalysts, the rate-limiting step is always the conversion of NO_3_ to NO_2ads_^−^.^37^ The *in situ* Fourier transform infrared spectroscopy in most studies is employed to explore the formation of hydroxylamine (NH_2_OH) during the NO_2_^−^/NO_3_^−^ reduction process, which is a vital feedstock for caprolactam synthesis and pesticide production.^38^ However, in most cases, NH_2_OH is inclined to be converted to NH_4_^+^.

### 2.2 Design principles of NO_*x*_^−^ reduction electrocatalysts

It is evident from the above that the selectivity and energy efficiency of NO_*x*_^−^ reduction can be influenced by the pH of the electrolyte and the reactor designs. In general, the required potential of NO_*x*_^−^ reduction in the alkaline environment is much lower than that in neutral and acidic environments.^39–41^ Alkaline conditions are more conducive to inhibiting the competing HER compared with acidic conditions providing more active hydrogen species. In neutral or alkaline environments, the required protons for NO_*x*_^−^ reduction are obtained from dissociation of water molecules. Diffusion of water molecules in neutral solutions is the rate-determining step of proton formation, while the rate-determining step in alkaline environments is the transport of adsorbed *OH. Intriguingly, the *OH produced facilitates the generation of NH_4_OH due to the attraction of nitrogenous species, which accelerates NO_*x*_^−^ reduction and reduces the competition of the HER. Thus, the design of NO_*x*_^−^ reduction catalysts for alkaline conditions is discussed in the majority of efforts available currently, since NO_*x*_^−^ reduction under alkaline conditions is easier to conduct with decent selectivity. In view of the neutral or acidic conditions of most wastewater, the associated design of catalysts under neutral and acidic conditions will also be discussed in the wastewater treatment section. In this section, the design principles of NO_*x*_^−^ reduction catalysts under alkaline conditions will be primarily discussed.

#### 2.2.1 Activating N-containing intermediates

**Fig. 3:**
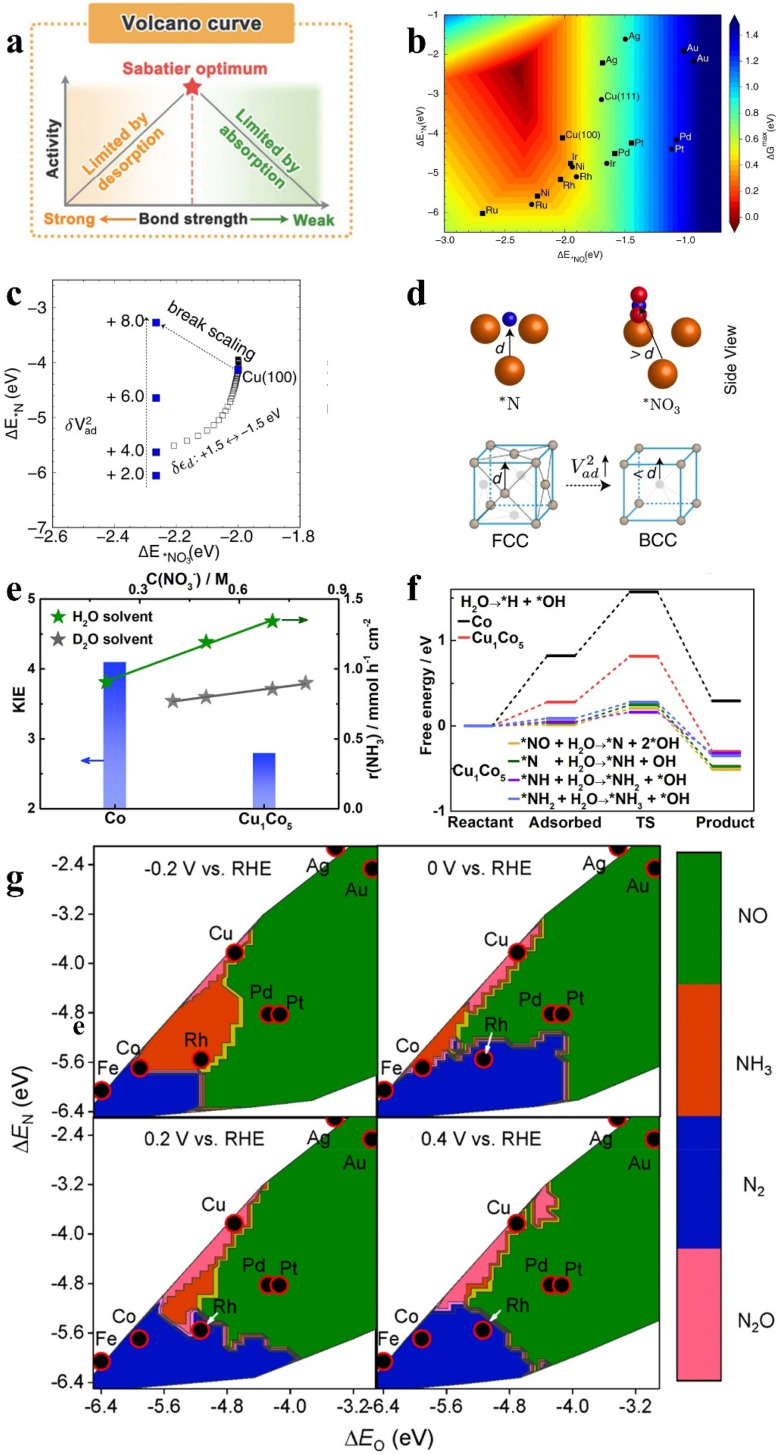
(a) Volcanic curves of activity based on Sabatier's principle. Reproduced with permission.^47^ Copyright 2022, Elsevier. (b) The activity volcano plot of various metal elements for the conversion of NO_3_^−^ to NH_3_, (c) adsorption energies of *NO_3_ and *N on Cu (100) under different *V*_ad_^2^. (d) The models of the (100) facet and (111) facet. Reproduced with permission.^48^ Copyright 2022, Springer Nature. (e) Reaction kinetics and H/D kinetic isotope effect of the NO_3_^−^RR with Cu_1_Co_5_ and (f) Gibbs free energies on Co and Cu_1_Co_5_. Reproduced with permission.^49^ Copyright 2023, American Chemical Society. (g) Theoretical selectivity maps to various nitrogen-containing products from electrocatalytic NO_3_^−^ reduction on the basis of Δ*E*_O_ and Δ*E*_N_ under different applied voltages. Reproduced with permission.^33^ Copyright 2019, American Chemical Society.

The ideal catalyst with competitive cost is expected to exhibit high selectivity and faradaic efficiency (FE) at low potentials, resulting in superior energy conversion efficiency and promising industrial application prospects.^42^ The negative potentials below −0.2 V are required in the majority of electrochemical systems reported for NH_3_ production, typically achieving unfavorable energy consumption (21–38 kW h kg^−1^).^43^ The key to reduce the overpotential and enhance the energy conversion efficiency lies in breaking the scaling relations between adsorption energy and activation energy during the reduction process.^44,45^ The scaling relationship is proposed on the basis of Sabatier's principle, which suggests a linear relationship between the adsorption energies of certain two reaction intermediates during the heterogeneous catalytic process.^46^ In addition, the relationship between the adsorption energy of a key intermediate and the reaction activity is exhibited in Fig. 3a. The excessively strong adsorption energy can result in catalyst surfaces that are densely covered with the intermediates, and exceedingly weak adsorption energy can prevent the reaction from proceeding.^50,51^ Therefore, appropriate modulation of adsorption energies of reaction intermediates can facilitate the reaction by promoting the adsorption of reactants and accelerating the desorption of products. In order to obtain a better description of the scaling relationship in the NO_*x*_^−^ reduction process, the activity volcano plot (Fig. 3b) has been constructed employing adsorption energies of the hollow *N and bridge-bidentate *NO_3_ as reactivity descriptors in NO_3_^−^ reduction.^48^ It can be observed from Fig. 3b that Cu is highly active for NO_3_^−^ reduction and the Cu (100) facet possesses more potential to break the scaling relationship compared to the (111) facet. As described in Fig. 3c, the adsorption strength of *N at the (100) hollow site increases with the enhancement of interatomic coupling strength (*V*_ad_^2^), which is caused by the obtained dominant position of Pauli repulsion with decreasing adsorbate-metal antibonding states. The advantage of the (100) facet is displayed in Fig. 3d compared to the (111) facet, and the stronger interatomic coupling can be achieved in the (100) facet due to a shorter distance between subsurface metal–ligand and *N. The hollow *N is more easily destroyed due to the dominant effect of Pauli repulsion, which promotes the hydrogenation of *N to form NH_3_. This interesting strategy provides inspiration for the construction of (100)-oriented B2 CuPd nanocubes to break the scaling relationship *via* modifying the Pauli repulsion between the metallic d-state and the adsorbate frontier orbital. The (100)-oriented B2 CuPd nanocubes have been confirmed to break the scaling relationship with increased *NO_3_ adsorption and attenuated *N binding. The enhanced bridge-bidentate *NO_3_ adsorption is caused by the upshift of the d-band center position in Cu after introducing Pd.

#### 2.2.2 Balancing nitrogen species and hydrogen species

In addition to activation of N-containing intermediates, H_2_O dissociation also deserves attention for reducing overpotential and enhancing energy efficiency. Since one NH_3_ requires six H_2_O molecules dissociated under alkaline conditions, the Cu_1_Co_5_ alloy is developed for NO_3_^−^ reduction, exhibiting the higher half-cell energy efficiency of 44.9% than those of Cu, Co, and other Co-based alloys.^49^ As demonstrated in Fig. 3e, it is suggested by the declined kinetic isotope effect in Cu_1_Co_5_ with more favorable NO_3_^−^ reduction activity that the bond to the isotopic atom is broken in the rate-determining step of NO_3_^−^ reduction.^52^Fig. 3f indicates the root of remarkable activity in Cu_1_Co_5_ under positive potentials, obtaining the beneficial dynamic equilibrium between active hydrogen generation and corresponding N-species consumption. The smaller energy barrier for H_2_O adsorption on Cu_1_Co_5_ indicates water molecules that are more easily adsorbed and dissociated on the alloy surface. The active hydrogen formation is difficult under positive potential,^53^ thus, H_2_O is directly involved in the N-species conversion process, which greatly hinders the HER side reaction.

Apart from the excellent energy conversion efficiency, high selectivity is also required in catalysts. The activity and selectivity of transition metals are exhibited in Fig. 3g for NO_3_^−^ reduction, which is predicted by exploring the adsorption energies of O and N atoms under different potentials.^33^ It is revealed that catalysts with moderate Δ*E*_O_ and Δ*E*_N_ are inclined to exhibit more remarkable NH_3_ selectivity at more negative potentials. The modulation of Δ*E*_O_ and Δ*E*_N_ can be achieved by adjusting the d-band center of the catalyst. For example, Cu50Ni50 was obtained by introducing Ni into Cu, which exhibited six-fold higher NO_3_^−^ reduction activity than pure Cu at the same potential.^54^ After the introduction of Ni, the d-band center of Cu50Ni50 was shifted by 0.28 eV, compared to the d-band center position of pure Cu (−2.84 eV). The regulation of the d-band center has been confirmed to contribute to modulating the adsorption energies of intermediates including *NO_3_^−^, *NO_2_, and *NH_2_, leading to the enhanced performance of Cu50Ni50 for NO_3_^−^ reduction.

**Fig. 4:**
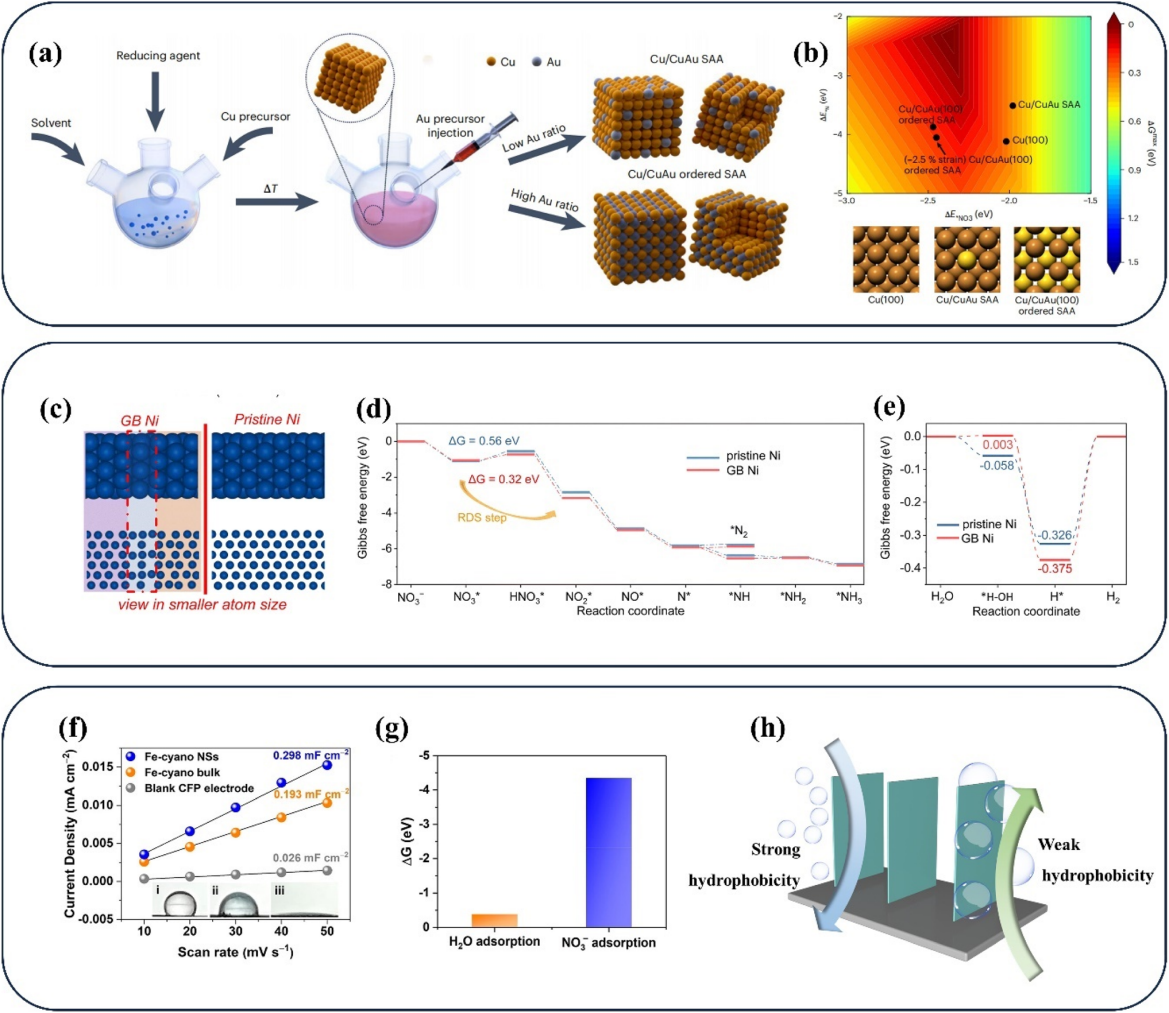
(a) Schematic diagram of synthesizing Cu/CuAu core/shell nanocrystals, (b) the binding energies of *NO_3_ and *N in related catalysts. Reproduced with permission.^57^ Copyright 2023, Springer Nature. (c) The structure model, (d) Gibbs free energy diagrams for NO_3_^−^ reduction (e) for the HER of pristine Ni and GB Ni. Reproduced with permission.^58^ Copyright 2023, Royal Society of Chemistry. (f) Contact angle measurement and (g) adsorption energy on Fe-cyano NSs. Reproduced with permission.^59^ Copyright 2022, American Chemical Society. (h) The states of bubbles on different aerophobic surfaces.

Moreover, reducing competition from other side reactions is also worthy of attention for NO_3_^−^ reduction in aqueous systems,^55^ including (1) the coupling reactions between N_ads_ and N_ads_ for blocking NH_3_ production and causing the generation of N_2_, N_2_H_4_, and N_2_O, (2) the competition for active hydrogen species with the HER only involving two-electron transfer. Single-atom metal-based catalysts offer new insights into reducing the direct coupling of N_ads_ in adjacent active sites due to the lack of adjacent sites,^56^ which is promising for inhibiting the generation of by-products including N_2_ and N_2_O and enhancing NH_3_ selectivity. In addition, the advantages of low metal loading and high metal utilization ratio in single-atom metal-based catalysts endow single-atom metal-based catalysts with the potential to be extremely cost-effective catalysts. Direct solution-phase synthesis (Fig. 4a) is employed for obtaining Cu/CuAu core/shell nanocrystals with tunable single-atom alloy layers.^57^ The synthetic Cu/CuAu nanocrystals reach a decent FE of 85.5% in NO_3_^−^ reduction at −0.5 V *vs.* RHE with high densities of single atoms. The weakened anchoring of N_ads_ (Fig. 4b) is conducive to the decent performance of Cu/CuAu nanocrystals, which is caused by strong repulsion from the gold ligand in subsurface or single-atom gold in the surface.

Furthermore, the NO_*x*_^−^ reduction process can be hampered by the existence of the HER, which is due to the competition for active hydrogen species^60^ and the blockage of active sites by the H_2_ generated.^61^ The relatively inert metals including Cu during the water splitting process have gained much attention in numerous studies,^62–64^ with a view to eliminating competition with the HER in NO_*x*_^−^ reduction. However, considering that the NO_*x*_^−^ reduction process relies heavily on the active hydrogen produced by H_2_O splitting, the blind inhibition of active hydrogen generation can hinder the subsequent hydrogenation process of the NO_*x*_ reduction process. In the common design strategy, the hydrogenation process of N species is promoted by lowering the energy barrier of *N formation when active hydrogen is abundant. Numerous efforts have employed diverse strategies such as doping and defects to lower the energy barrier of N_ads_ formation,^65,66^ thus promoting the subsequent hydrogenation processes. Ni nanoparticles with grain boundary defects have been developed for opening up an entirely new strategy of utilizing water splitting and preventing H_2_ formation.^58^ Abundant active hydrogen species can be generated in the catalytic process due to the decent HER activity of Ni. Besides, the formation of H_2_ is difficult on the surface owing to the strong retention capacity of active hydrogen species in grain boundary (GB) regions (Fig. 4c). With the presence of grain boundary regions, the active hydrogen species are transferred to the neighbouring adsorbed intermediates for accelerating NO_*x*_^−^ reduction. The Gibbs free energy diagrams of GB Ni for NO_3_^−^ reduction and the HER are exhibited in Fig. 4d and e. The excellent selectivity of GB Ni for NH_3_ is confirmed by the lower formation energy of the N–H bond than that of N_2_ in Fig. 4d. The stronger retention capacity of GB Ni for active hydrogen species is evidenced by the higher energy barrier for obtaining H_2_ (Fig. 4e). In addition, the energy barrier for the conversion of *NO_3_ to *NO_2_ in the GB region is lower than that of forming H_2_, confirming that this strategy facilitates the inhibition of the HER, where the active hydrogen species tends to reduce *NO_3_ rather than form H_2_. The above strategy employing two sites has opened up a new avenue for achieving sufficient active hydrogen species and excellent NH_3_ selectivity. In this strategy, the production of protons is promoted in one site acting as a proton warehouse, while the active hydrogen species are stored temporarily at the other site to facilitate subsequent hydrogenation processes.

#### 2.2.3 Focusing on hydrophilic and aerophobic properties

In addition, the hydrophilic and aerophobic properties of the catalyst surface are also deserving of emphasis.^67–69^ In view of the blockage of NO_*x*_^−^ reduction active sites caused by the by-products including N_2_ and H_2_, the ideal NO_*x*_^−^ reduction catalysts under aqueous conditions should possess strong aerophobic properties along with high affinity for aqueous electrolyte. The strongly hydrophilic surface of catalysts is more conducive to the adsorption of reactants. For example, Fe-based cyano-coordination polymer nanosheets (Fe-cyano NSs) can achieve an excellent NH_3_ rate up to 15.49 mmol h^−1^ cm^−2^ with FE exceeding 90% at −0.5 V *vs.* RHE.^59^ The remarkable performance can be attributed to the super-hydrophilic surface of Fe-cyano NSs (Fig. 4f), which is conducive to enhancing the contact between the electrolytes and the electrode surface for faster conversion to Fe^0^ active sites, facilitating adsorption of NO_*x*_^−^ ions (Fig. 4g). The NO_*x*_^−^ reduction process is inevitably accompanied by side reactions producing gases such as H_2_ and N_2_. Thus, enhancing the aerophobicity of the electrode interface is essential for achieving decent reaction efficiency.^70^ As depicted in Fig. 4i, bubbles with smaller diameters are more favourable for mass transfer processes in catalysts with a stronger aerophobic nature, eliminating the accumulation of bubbles and strong adhesion on the electrode surface caused by larger bubbles.^71–73^

It is seen above that a series of hurdles are present in NO_*x*_^−^ reduction catalysts including inferior selectivity and unpromising energy conversion efficiency. In order to achieve a sustainable and competitive electrochemical ammonia production route, the following points could be considered for constructing outstanding NO_*x*_^−^ reduction electrocatalysts: (1) the enhanced adsorption of NO_*x*_^−^ can promote the reduction reaction, but accompanied with the dilemma of difficult product desorption. The key to solving the problem for significantly improving performance lies in breaking the scaling relationship between the adsorption energies of intermediates and reactants; (2) the competition with the HER is difficult to escape for NO_*x*_^−^ reduction under aqueous conditions. Boosting NH_3_ selectivity and reducing H_2_ formation can be achieved by employing appropriate strategies utilizing water splitting for achieving sufficient supply of active hydrogen species and hindering direct coupling of active hydrogen species; (3) the stability of intermediates including *NO_2ads_ or *NOH_ads_ should not be ignored for more efficient NH_3_ production, which can decrease other side reactions generating N_2_ or NO; (4) the hydrophilic and aerophobic properties on the catalyst surface are also deserving of emphasis. The strongly hydrophilic and aerophobic surface of catalysts is more conducive to the adsorption of reactants and circumventing blocked active sites.

### 2.3 Reactor design

**Fig. 5:**
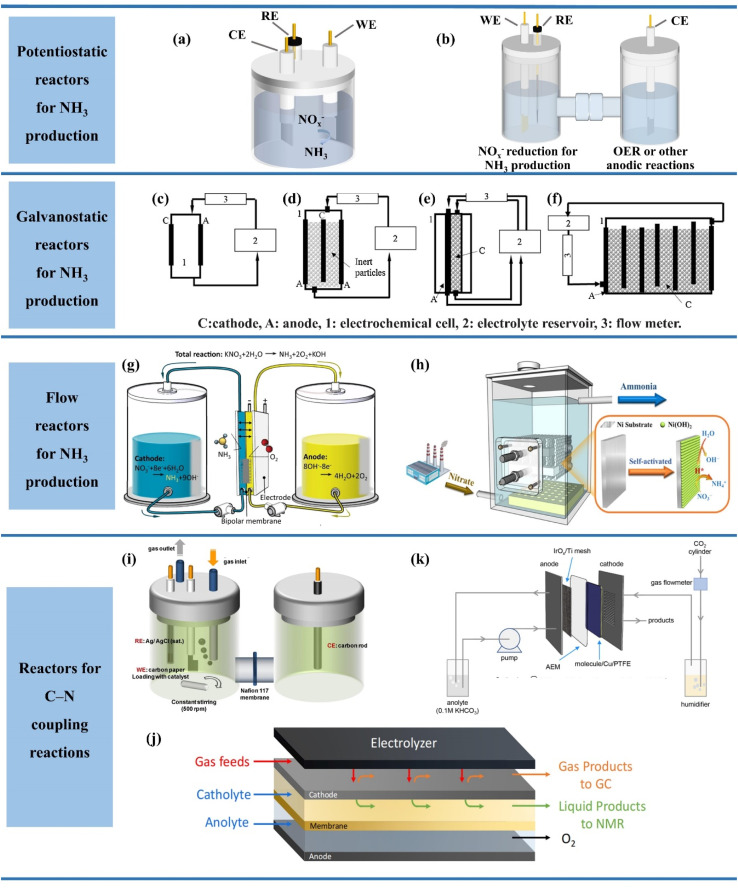
(a) Single-chamber and (b) dual-chamber reactors, (c) plate electrode cell, (d) fluidizing bed of the inert particle cell, (e) packed bed cathode cell, (f) vertical moving particle bed cell. Reproduced with permission.^74^ Copyright 2018, Elsevier. (g) Continuous NH_3_ electrosynthesis with a bipolar membrane reactor and (h) electrochemical reactor in a pilot scale used in electrocatalytic reduction of NO_3_^−^/NO_2_^−^. (i) H-cells, (j) membrane-based flow reactors and (k) membrane electrode assembly electrolyzers for C–N coupling reactions. Reproduced with permission.^75^ Copyright 2023, Elsevier.

The current reactor designs under different scenarios are summarized in Fig. 5. The most common reaction devices available in the current laboratory are shown in Fig. 5a and b, which can be divided into single-chamber cells and dual-chamber cells (H-type cells). The difference between the single-chamber reactor and dual-chamber reactor is caused by the ion exchange membrane and electrode spacing. The single-chamber reactor possesses the advantages of smaller internal resistance, more simplified design and lower cost than the double-chamber reactor due to the absence of an ion exchange membrane and smaller electrode spacing.^76^ However, it is difficult to avoid that the dissolved metal ions generated by the anode may also be deposited on the catalyst surface in a single-chamber reactor, thus reducing the FE of ammonia production.^77^ Therefore, two chambers separated by ion exchange membranes are commonly employed in experimental studies to form a dual-chamber reactor. In spite of the higher ohmic resistance and larger energy consumption, the double chamber reactor can substantially decrease the occurrence of side reactions and enhance the FE of ammonia production. Notably, the dual-chamber reactors employed in a majority of the studies are typically operated under constant voltage. The dual-chamber reactor may not be more advantageous than a single-chamber reactor when the “multi-potential steps” are employed. For example, the *in situ* reconstruction of the Cu surface during NO_3_^−^ reduction was achieved by pulsed electrolysis of the Cu electrode.^78^ Both the anodic pulsed oxidation reaction of Cu and the cathodic pulsed nitric acid reduction reaction occur in a single compartment, with the possibility of reoxidation of the reduction intermediates during the anodic pulse. In the process of electrolysis, the Cu oxidation by the anodic pulse and NO_3_^−^ reduction by the cathodic pulse occurred in the same chamber, where the re-oxidation of reducing intermediates may occur during the anodic pulse. In this process, the selection of single-chamber reactors is caused by the unavoidable intermediate crossover in dual-chamber cells.

Nevertheless, the reactor systems employed in experimental studies are still restricted for industrial applications. The three-electrode system (potentiostatic electrolysis) often employed in experimental studies tends to result in slower nitrate reduction owing to higher activation energy in comparison to galvanostatic electrolysis.^74^ In addition, the struggle of the three-electrode system for large-scale application in wastewater treatment is also attributed to the complex power supply conditions.^79^ Consequently, the galvanostatic system without the involvement of reference electrodes for control is more promising for industrial applications, but it is worth noting that the required constant applied voltage should be evaluated in advance when using galvanostatic electrolysis to suppress side reactions and maximize economic efficiency.

The designs of the reactors for galvanostatic electrolysis are displayed in Fig. 5c–f, which are more favourable for industrial applications due to the requirement of only two electrodes in the electrolyser containing the cathode and anode. The designed reactors are composed of three components, including the electrochemical cell, electrolyte reservoir and flow meter. The simplest reactor (Fig. 5c) is shown with one anode and one cathode in the electrochemical cell, which contributes to the decreased solution resistivity and reduced operating cost due to the small spacing between the pole plate electrodes. However, the mass transfer process is not desirable enough in Fig. 5c. In order to accelerate the mass transfer, the reactor (Fig. 5d) is designed with various turbulence promotors or fluidized bed inert particles in the inter-electrode space. In addition, NO_*x*_^−^ reduction can be facilitated due to the production of reduction promoter H_2_ when hydrogenated catalysts are employed as fluidized particles. Moreover, the further optimization of reactors is achieved in Fig. 5e and f with enhanced active area and faster mass transfer, which is caused by the packed bed cathode cells.

The reactor volume in the laboratory can hardly exceed 500 mL, which makes it extremely difficult to meet the industrial demands and achieve treating large quantities of wastewater.^11^ In order to achieve uniform NO_*x*_^−^ reduction, the device is operated in continuous flow state. In continuous flow reactors, more challenges are imposed on electrode design, hydraulic flow state and ion-exchange membrane. The inferior hydraulic flow state can be enhanced by employing the strategies of improving reactor configuration including spiral tube reactors. Obviously, more attention is being paid to electrode design^80^ and the improvement of the ion-exchange membrane.^81^

In terms of electrode design, reasonable electrode space and sufficient active sites are required to achieve accelerated mass transfer and decent reduction efficiency. Considering the technical difficulties and increased internal resistance of electrodes associated with directly enlarging the electrodes, the formation of an electrode module by multiple electrode sheets provides a reasonable solution in Fig. 5h.^82^ The electrode module assembly is placed in a continuous flow reactor, which can overcome the drawback of insufficient reaction sites on the electrode surface and alleviate the limited treatment capacity of intermittent reactors.

In addition, ion-exchange membranes are also essential for electrochemical NH_3_ synthesis, being required to isolate the asymmetric electrolytes on two compartments and inhibit the re-oxidation of NH_3_ diffused to the anode. Compared with the severe ion crossover in the unipolar ion-exchange membrane, a bipolar membrane (BM) has been proposed with a mortise–tenon joint interlayer (Fig. 5g), which is composed of an anion exchange layer and a cation exchange layer.^83^ In this modified bipolar membrane, ion selectivity is formed by electrostatic repulsion of the bipolar membrane. The total dissociation rate and the stability of the bipolar membrane are improved due to the increased hydrolysis dislocation sites. The electrolytic ammonia production device assembled with this membrane lays the foundation for the achievement of continuous and stable electrochemical ammonia synthesis at high current density exceeding 1000 mA cm^−2^.

As for the electrochemical C–N coupling reactions based on NO_*x*_^−^ reduction, some differences will exist in the design of the reaction equipment due to the injection of gases involved in some C–N coupling reactions. Three types of electrochemical reactors are illustrated in Fig. 5i–k demonstrating potential for application in electrochemical C–N coupling reactions, which consist of an H-type cell, membrane-based flow reactor and membrane electrode assembly (MEA) electrolyser.^75^ The H-type cell is the most commonly employed electrochemical reactor for C–N coupling reactions in laboratories currently due to its low cost and easy installation.^84^ The most obvious difference between the H-type cell for C–N coupling reactions and that for NO_*x*_^−^ reduction is the increase of gas inlet and outlet ports. The gaseous reactants including CO_2_ in the H-type cell are dissolved in aqueous solution and then diffuse to the interface between the cathode electrolyte and the working electrode. The concentration of gaseous reactants on the cathode surface is lowest due to the distance required for diffusion to the cathode in this process. In addition, the H-type cell is also still restricted for further industrial application due to its inferior mass transfer and high electrical resistance.

Membrane-based flow reactors (Fig. 5j) employing gas diffusion electrodes (GDEs) as cathodes hold promise for overcoming the drawbacks of the H-electrolyser, which possesses a continuous flow of electrolyte in both anode and cathode compartments.^85^ Due to the presence of GDEs, the gaseous reactants can enter the cathode directly instead of diffusing from the cathode electrolyte. Consequently, the presence of GDEs ensures sufficient supply of gaseous reactants near the catalyst surface to allow the reaction to proceed at high current density. However, the membrane-based flow reactor is also hampered by poor stability, which is mainly attributed to hindered transport of gaseous reactants caused by electrolyte penetration in GDEs during the electrolysis process.

The emerging MEA electrolyzer provides novel insights into circumventing the poor stability of the membrane-based flow cell, which eliminates the cathode fluid based on the membrane-based flow cell and employs dampened reactant streams as feeding gases (Fig. 5k).^86^ The elimination of the cathode electrolyte facilitates enhanced stability and energy efficiency of the electrochemical system, which is caused by reducing the ohmic resistance and bypassing the poor stability of GDEs due to the penetration of the electrolyte. In addition, compared to products dissolved in the electrolyte of the membrane-based flow cell, products obtained by the MEA electrolyser remain in the gaseous phase, which is easy to collect by condensation for dramatically reducing the cost of separating the product. In spite of the unexplored application of the MEA electrolyser in C–N coupling reactions currently, it has provided a promising approach to achieve ultrastable C–N coupling reactions at high current density.

## 3. Wastewater treatment

Enormous efforts have been made towards the removal of NO_3_^−^/NO_2_^−^ in underground water for restoring the global nitrogen cycle. Public health is directly threatened by NO_*x*_^−^ ions which impair oxygen transport and trigger the “blue baby syndrome”. More importantly, the destroyed ozone layer and worsened global warming can be caused by N_2_O generated through bacterial denitrification in nature.^87^ The electrochemical NO_*x*_^−^ reduction has gained much attention as a promising denitrification alternative, which possesses the advantages of mild operating conditions, no deleterious residues and small installation footprint. Although the NO_*x*_^−^ reduction to NH_3_ pathway has demonstrated more benefits than NO_*x*_^−^ reduction to N_2_ owing to the easier formation of the N–H bond and higher application value of NH_3_, it is worth noting that employing the NO_*x*_^−^—NH_3_ approach for treating wastewater has also been doubted due to the following two reasons: (1) the uncompetitive economic benefits due to insufficient NO_*x*_^−^ concentration;^88^ (2) the difficult extraction of dissolved NH_3_ causing worse environmental implications.^89^ Thus, the treatment of wastewater containing nitrate/nitrite is recommended in many efforts to convert low-concentration NO_*x*_^−^ ions into N_2_ or recycle high-concentration NO_*x*_^−^ ions to other N-containing fertilizers.^90–92^ For achieving the optimal balance between efficiency and economy in large-scale wastewater treatment employing the NO_*x*_^−^—NH_3_ approach, it is necessary to delve into electrocatalyst development, actual wastewater composition, reaction system design and product separation.

### 3.1 Factors influencing electrochemical NO_*x*_^−^ reduction

In view of the complex composition of wastewater, several studies have explored the influences of ion concentration, other existing inorganic ions, and pH on NO_*x*_^−^ reduction in wastewater to facilitate more efficient utilization of wastewater streams for ammonia production.^93–95^ The concentrations and species of ions in some common wastewater sources are illustrated in Table 1. Indeed, NO_2_^−^ reduction to NH_3_ is thermodynamically and kinetically facile, making it promising for NH_3_ synthesis. Despite the mass of NO_2_^−^ in wastewaters is far less than that of NO_3_^−^ (Table 1), NO_3_^−^ ions in wastewater are always transformed to NO_2_^−^ by micro-organisms. With respect to the effects of other ions, it is obvious that NO_3_^−^ in sufficiently large concentrations is far more competitive than other ions, and thus the reaction rate is hardly affected.^100^ However, such wastewater is not especially common and therefore concentrated NO_3_^−^-containing wastewater offers greater potential for ammonia production. The effects of pH and other inorganic ions on the reaction rate of the nitrate reduction process should not be neglected. The NO_*x*_^−^ reduction reaction can be conducted over a broad range of pH; however, the performance and selectivity of NO_*x*_^−^ reduction differ dramatically at different pH conditions. In addition, the drastic changes in the pH of the electrode interface and solution probably cause altered reaction mechanisms during the reduction process with proton depletion or ammonium hydroxide production.^101^ Thus, buffer solutions with high concentrations are employed to mitigate drastic changes of pH in the majority of studies on electrochemical NO_*x*_^−^ removal. The dominant HER under acidic conditions can diminish the selectivity and FE of NO_*x*_^−^ reduction. Despite the fact that the selectivity of NO_*x*_^−^ reduction can be enhanced under alkaline conditions, the reduction kinetics can be boosted with the presence of indirect catalytic processes in acidic environment. It has been confirmed that NO_2_^−^ is the dominant product for NO_3_^−^ reduction in an alkaline environment, and the generation of NH_3_ can be accelerated with the increase of proton concentration.^11^

**Table 1:** Available NO_3_^−^/NO_2_^−^-rich wastewater streams

Type of wastewater	pH	Main composition	NO_3_^−^ concentration	NO_2_^−^ concentration	Ref.
Textile wastewater	Neutral	NO_3_^−^, Cl^−^	7.4 mM	—	96
Industrial wastewater	Alkalescent	NO_3_^−^, NH_4_^+^, Cl^−^	41.6 mM	—	97
Polluted ground water	Unknown	NO_3_^−^, NO_2_^−^, NH_4_^+^	0.88–1.26 mM	0.22–1.27 mM	98
Low-level nuclear wastewater	Alkaline	NO_3_^−^, NO_2_^−^, SO_4_^2−^, CO_3_^2−^, Cl^−^, F^−^, SiO_3_^2−^, CrO_4_^2−^	1.95 M	0.55 M	99

For inorganic ions, the impacts on nitrate reactions are diverse, including positive and negative effects. It is found that alkali metal cations follow the order of Li^+^ < Na^+^ < K^+^ < Cs^+^ to enhance the rate of NO_3_^−^ reduction.^11^ The cations weaken the repulsive force between the negative ions and the cathode and facilitate the reduction of NO_3_^−^ on the cathode since the cations modify the bimolecular structure of the cathode and form transient neutral ion pairs.^102^ As for multivalent cations, the presence of NH_4_^+^, Ca^2+^ and La^3+^ can achieve higher rates than alkali metal cations, but some cations such as Ca^2+^ and Mg^2+^ can be adsorbed on the cathode surface to form precipitates, resulting in poisoning of the cathode active sites and reduced reaction rates.^103^ Similarly, the reduction reaction of NO_3_^−^ is also affected by anions. The negative effect of anions on the reaction rate is caused by the competition of anions for adsorption sites. The anions are ranked in the order of I^−^ > Br^−^ > Cl^−^ > F^−^ to reduce the rate of nitrate reduction.^104^

### 3.2 Reactors for NO_*x*_^−^ removal and product collection

**Fig. 6:**
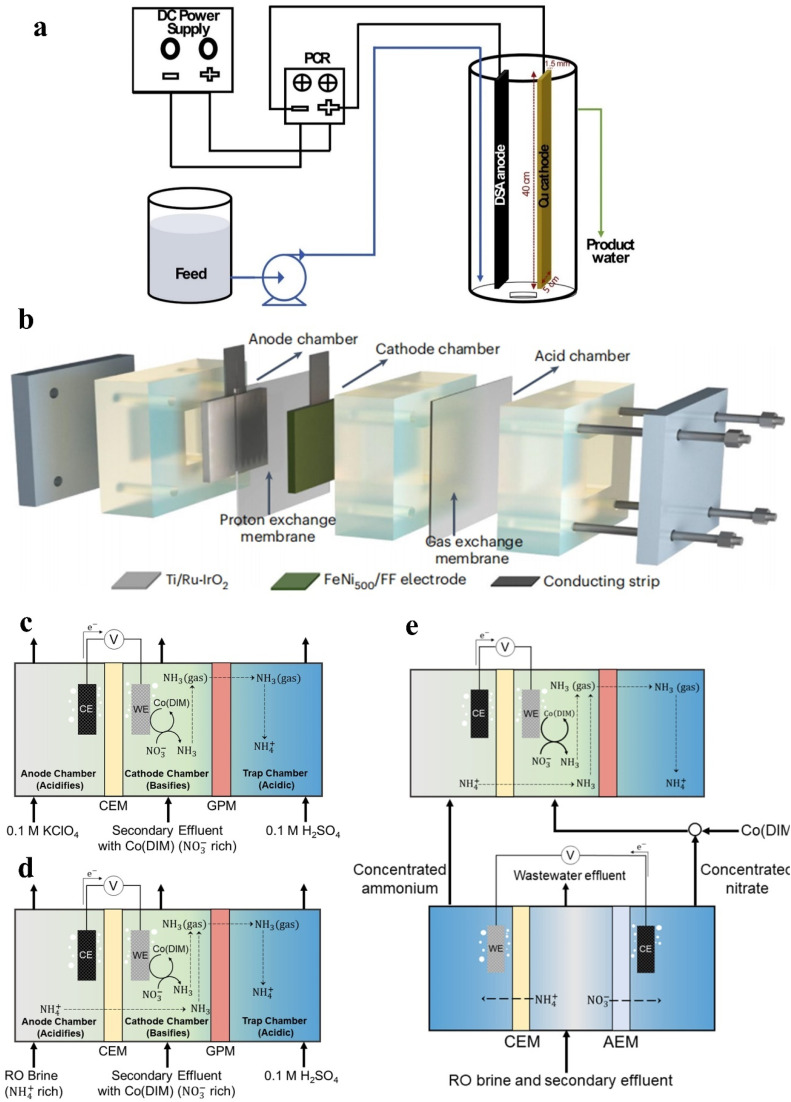
(a) Electrochemical continuous flow system. Reproduced with permission.^106^ Copyright 2020, Elsevier. (b) Schematic of the three-chamber membrane distillation reactor. Reproduced with permission.^5^ Copyright 2023, Springer Nature. (c) The ECS reactor, (d) ECS reactor with NH_4_^+^-rich RO brine and (e) ECS reactor with ED. Reproduced with permission.^107^ Copyright 2023, American Chemical Society.

The continuous flow state is usually adopted in the operation of industrialized reactors. In spite of the preliminary discussion of continuous flow reactors in Section 2.2, the feasibility of reactors is of significant importance for enhancing the scale and commercial value of wastewater treatment.^105^ Regretfully, the feasibility of continuous flow reactors has rarely been explored for removing NO_*x*_^−^ ions in current studies. Makover *et al.* have explored the feasibility of Cu-dimensionally stable anode (DSA) electrodes in a continuous flow reactor (Fig. 6a) for treating sewage after Donnan dialysis.^106^ The anode (DSA) employed is Ti covered with RuO_2_/IrO_2_. In Donnan dialysis, the NO_*x*_^−^ ions in sewage will transfer to the receiver compartment from the feed compartment due to electroneutrality induction, accompanied with the transfer of high concentration Cl^−^ and SO_4_^2−^ ions into the receiver driven by a concentration gradient. The excellent results employing Cu-DSA electrodes are obtained in high salinity NO_*x*_^−^ contaminated solution generated by Donnan dialysis. Optimal NO_3_^−^ removal can be reached at a low current density of 10 mA cm^−2^ and short residence time of 90 min, reaching 63% in high salinity Na_2_SO_4_ and 44% in high salinity NaCl. The promotion effect of high salinity SO_4_^2−^ ions and the inhibition effect of high salinity Cl^−^ ions on nitrate removal have also been confirmed.

In the current studies of separating products, converting NH_3_ to (NH_4_)_2_SO_4_ by coupling acid adsorption is a reasonable route. Fig. 6b exhibits a three-chamber membrane distillation reactor by coupling electrocatalysis with acid absorption.^5^ The electrodes involved in Fig. 6b are generally self-supported electrodes with active sites that fail to be fully utilized, compared with the homogeneous molecular catalyst achieving precise atomic coordination between reactants and catalytic active sites. Thus, a series of electrochemical stripping (ECS) reactors are developed using homogeneous molecular catalyst Co(DIM) for degrading contaminants and extracting products in large-volume and nitrate-rich wastewaters (typically <4 mM).^107^ The ECS recirculating batch process is shown in Fig. 6c. A cation exchange membrane (CEM) is used to avoid NO_*x*_^−^ diffusion from the cathode chamber to the anode compartment. During the reduction process, the increased pH of the catholyte is expected to exceed the p*K*_a_ of NH_3_ (9.25), causing the majority of products to be in the form of volatile NH_3_. Thus, a hydrophobic breathable membrane is utilized for dispersing volatile NH_3_ from the cathode chamber. The operation for 42 h in this reactor witnessed 70.5% NO_*x*_^−^ removal, which makes the treated water meet the drinking water limit. Moreover, the NH_3_ selectivity remains over 98.5% throughout this period which confirms that the Co(DIM)-mediated NO_*x*_^−^ reduction is rarely influenced by wastewater constituents.

In order to reduce electrical energy consumption and enhance the ammonia recovery rate, two additional process configurations are explored in Fig. 6d and e. In a parallel feed configuration (Fig. 6d), the KClO_4_ solution is replaced by NH_4_^+^-rich reverse osmosis (RO) brine, enhancing the NH_3_ recovery rate by 17 times and halving electrical energy consumption. An electrodialysis (ED) cell is added in Fig. 6e for concentrating NO_3_^−^ and NH_4_^+^ to provide a greater driving force for reaction and separation, which effectively enhances the rates of NO_3_^−^ removal and NH_3_ recovery by 10 and 95 times, respectively. Moreover, Co(DIM) is added in the concentrated NO_3_^−^ solution instead of wastewater in this ED-concentrated parallel configuration for preventing catalysts from separating from the wastewater.

### 3.3 Catalyst design for large-scale wastewater treatment

**Fig. 7:**
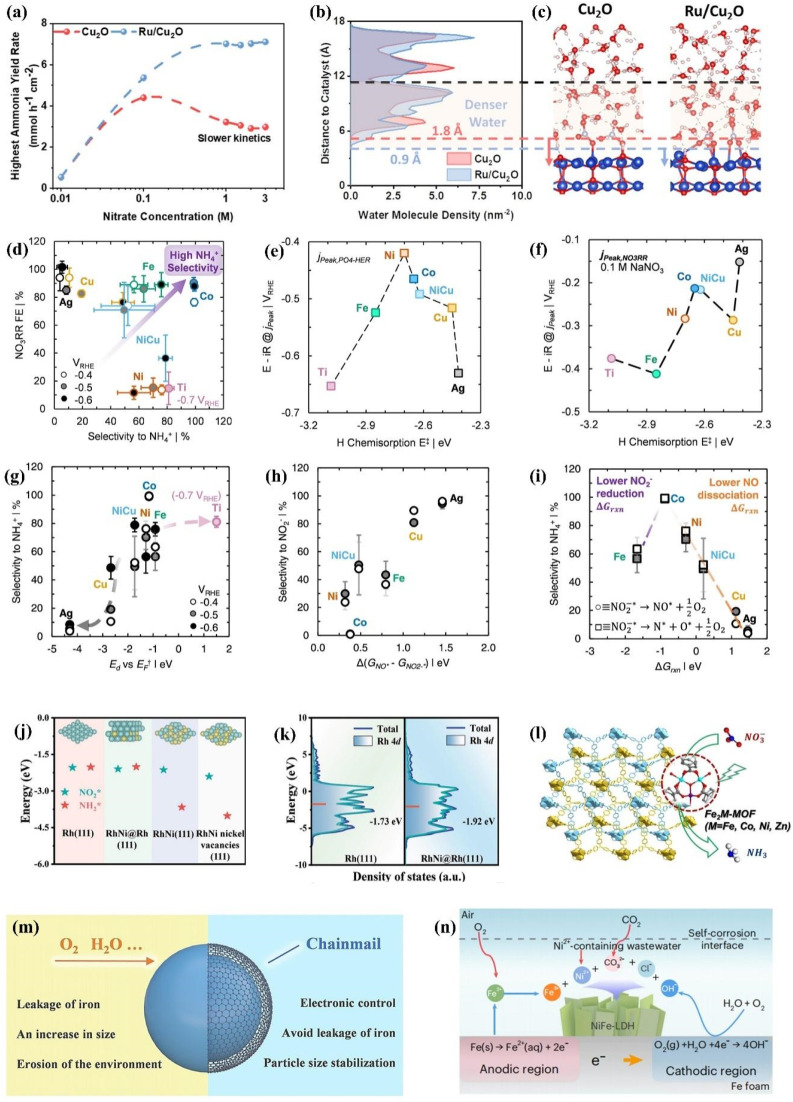
(a) Optimized NH_3_ yield rate of various catalysts in an electrolyte containing 1 M KOH and different NO_3_^−^ concentrations and (b) densities of water molecules, and (c) representative molecular dynamics simulation snapshots. Reproduced with permission.^111^ Copyright 2024, American Chemical Society. (d) NO_3_^−^ reduction FE and selectivity on different metals. Relationships between the H chemisorption energy and potential in mass-transfer limited (e) phosphate-mediated HER and (f) NO_3_^−^ reduction in 0.1 M NaNO_3_. (g) Relationship between selectivity and *E*_d_*vs. E*_F_. Relationship between reaction free energies (Δ*G*_rxn_) for converting *NO_2_^−^ to *NO and selectivity to (h) NO_2_^−^ and (i) NH_4_^+^. Reproduced with permission.^112^ Copyright 2022, American Chemical Society. (j) Adsorption energy of 
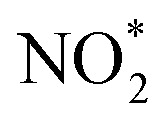
 and 
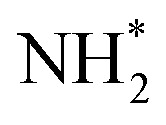
 and (k) d-band centers of different catalysts. Reproduced with permission.^113^ Copyright 2024, Wiley-VCH. (l) 3D framework of Fe_2_M-MOF. Reproduced with permission.^114^ Copyright 2023, Wiley-VCH. (m) The role of graphene nano-chainmail. Reproduced with permission.^115^ Copyright 2023, Wiley-VCH. (n) The specific reactions of the self-corrosion process. Reproduced with permission.^5^ Copyright 2023, Springer Nature.

For industrial treatment of NO_*x*_^−^-containing wastewater, it is necessary to take into account the concentrated electrolyte, the pH of electrolytes and the electrode amplification for improving the economic viability of electrosynthesis.^107–110^ Currently, the NO_*x*_^−^ concentration is typically lower than 1 M in a majority of efforts. Fig. 7a shows the nonmonotonic change of NH_3_ yield on Cu_2_O from 0.01 to 3 M NO_3_^−^. In the dilute regime (0.01 to 0.1 M), the production rate increases with nitrate concentrations due to promoted mass transfer and reaction kinetics. However, a decline is experienced in the range from 0.1 to 3 M, which is caused by the excessive NO_3_^−^ adsorption for reducing water molecule adsorption on surface sites. To solve the dilemma of mismatched reaction kinetics between the HER and NO_3_^−^ reduction, Ru is introduced in Cu_2_O for more efficient NO_3_^−^ reduction.^111^ The water density profiles from the Cu_2_O and Ru/Cu_2_O surface are exhibited in Fig. 7b and c through molecular dynamics simulations. The higher H_2_O density in the Ru/Cu_2_O model is more conducive to promoting the collision between NO_3_^−^ ions and H_2_O molecules for more efficient hydrogen transfer, which endows Ru/Cu_2_O with 89% NH_3_ FE under 9.9 A for 3 M NO_3_^−^ reduction.

As for the amplified synthesis of electrodes, the electrode pieces are often present individually on a laboratory scale, so if they were to be scaled up directly, not only would there be modification, but also the internal resistance would increase. The direct amplification of electrodes faces the challenges of technical limitation for processing and sharp increases in resistance; the stacked electrode module mentioned in Fig. 5h provides a decent solution for avoiding the limitations induced by direct electrode amplification. Despite the extremely wide range of pH values in sewage collected, the majority of sewage is in neutral and acid environments. The catalysts discussed in Section 2 are designed for alkaline conditions; the HER competition will be more intense in neutral and acidic media than that in alkaline media.

As for the catalysts in a neutral environment, a series of transition metals and alloys have been explored including Ti, Fe, Co, Ni, Cu, Ag and Ni_0.68_Cu_0.32_, employing 0.1 M Na_*x*_H_3−*x*_PO_4_ solution to simulate the neutral environment.^112^ The promising catalysts have been screened under neutral conditions with exploration of the associated thermodynamic and kinetic parameters of catalysts. As demonstrated in Fig. 7d, metallic Co exhibits promising NO_3_^−^ reduction FE and selectivity for NH_3_ formation. Co with moderate H chemisorption energy (Fig. 7e and f) shows smaller cathode mass transfer limiting the nitrate reduction potential compared with transition metals binding H strongly such as Fe and Ti, indicating that strong H chemisorption energy leads to sluggish proton-coupled electron transfer and hydrogenation kinetics in NO_3_^−^ reduction. Further explorations of the origin of the excellent Co activity are demonstrated in Fig. 7g–i by probing the d-band center energy (*E*_d_) and calculated reaction free energies of NO_2_^−^ reduction to NO and further dissociation. The theoretical calculations have confirmed that more negative Δ*G*_NO_ads__ can be achieved when *E*_d_ approaches the Fermi level (*E*_F_), which is caused by the increasingly unoccupied antibonding molecular orbital formed between NO_ads_ and the catalyst surface. However, as illustrated in Fig. 7g, Co exhibits superior selectivity beyond 95% over a broad range of potentials, which significantly exceeds the selectivity of metals (Ni, Fe) with similar *E*_d_*vs. E*_F_. The calculated reaction free energies of NO_2_^−^ reduction to NO and further dissociation (Fig. 7h and i) are considered for better exploring the origin of the extraordinary NH_3_ selectivity of Co. As described in Fig. 7h, the selectivity for NO_2_^−^ decreases roughly with the reduced difference between ΔG_*NO_ and ΔG_*NO_2_^−^_. In spite of the lower NO_2_^−^ reduction energy barrier in Ni compared to Co, Ni exhibits inferior NH_3_ selectivity due to weaker decomposition for subsequently produced NO. In contrast, the lower NH_3_ selectivity of Fe possessing more favourable NO dissociation is caused by adverse NO_2_^−^ reduction activity. The volcanic trend of selectivity is described in Fig. 7i based on NO_2_^−^ reduction activity and NO dissociation energy of metals explored. An ideal state of catalysts with promising NH_3_ selectivity can be represented by Co with sufficiently strong tendency for NO binding and dissociation and adequate activity for NO_2_^−^ reduction.

The design of excellent catalysts can be conducted by approaching a series of parameters on Co including hydrogen affinity and *E*_d_*vs. E*_F_. For example, in spite of high hydrogen binding energy of the NiFe alloy for unfavorable NO_3_^−^ reduction, the NiFe alloy possesses *E*_d_*vs. E*_F_ and work function similar to Co, which cause better NO_3_^−^ reduction activity and selectivity than the mono-component metals (Ni, Fe). On the flip side, the performance of Co can also be further improved including the possible improvement of the energy conversion efficiency. Enhancing NO_3_^−^ affinity by pairing of metal oxides and electrolyte modulation are both effective approaches for boosting the NO_3_^−^ reduction activity of Co.

As for the catalysts under acidic conditions, there are major challenges including (1) stronger HER competition and (2) drastically reduced stability caused by the dissolution of metal catalysts in strong acidic environments.^110,111^ However, several advantages are also presented in acidic environments, which can avoid the subsequent extraction process and spillage loss of aqueous NH_3_ owing to the direct generation of nitrogen fertilizers such as (NH_4_)_2_SO_4_ and NH_4_Cl under acidic conditions.^112^ In order to resist acid-induced corrosion, Rh has recently been employed to construct an electrocatalyst and is one of the few metals that can withstand aqua regia. The RhNi@Rh bimetallenes are synthesized for weakening the adsorption of 
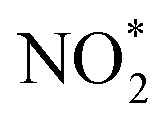
 and 
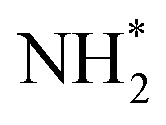
 to resist adsorption-induced Rh dissolution (Fig. 7j), which exhibit a declined d-band center (Fig. 7k) due to the compressive stress induced from the inside out by the RhNi alloy core. With the modified adsorption behavior of Rh, the catalyst demonstrates exceptional stability over an extended 400 h test in acidic environments.

The immobilisation also provides a plausible strategy for enhancing the stability of transition metal catalysts capable of effectively inhibiting the HER. Fe_2_Co-MOF (Fig. 7l) is obtained by assembling Fe_2_Co clusters and H_4_TPBD ligands,^115^ which bypasses the decreased catalytic efficiencies due to saturated metal centres in the majority of MOFs. Fe_2_Co-MOF exhibits superior electrocatalytic stability up to 75 h at −1.1 V *vs.* RHE in pH = 1 electrolyte, accompanied by NH_3_ yield approaching 20 653.5 μg h^−1^ mg_site_^−1^ and FE of 90.55%. The decent activity of Fe_2_Co-MOF derives from the suppressed HER and high turnover adsorption of NO_3_^−^ due to the unsaturated metal sites induced by trinuclear clusters. In addition, the catalytic efficiency is further enhanced in acidic environments filled with protons, since the transfer of electrons and reactants can be promoted by the redox-active dinitrogen ligand. The remarkable stability of Fe_2_Co-MOF can be primarily ascribed to the highly connected structure with robust coordinative bonds formed by the combination of high-valence Fe^3+^ and carboxylate ligands.

In addition to the approach mentioned above, armoured catalysts and self-corrosion reconstruction strategies also deserve to be utilized for enhancing long-term stabilities in the acid environment. Fig. 7m depicts the merits of armoured catalysts for NO_3_^−^ conversion to N_2_ protected by ultrathin graphene nanolayers, which could provide more insights for enhancing the stabilities of NO_*x*_^−^ reduction catalysts.^115^ Moreover, novel insights on expanding electrocatalyst and wastewater treatment can be provided by the economical self-corrosion approach (Fig. 7n) utilizing heavy metal ions in wastewater (Ni^2+^, Co^2+^ and Zn^2+^) for inducing the Fe surface to generate LDH nanosheets.^5^ This corrosion strategy is conducive to generating the active phase and avoiding conventional corrosion passivation. Furthermore, the contact between active sites and NO_*x*_^−^ is facilitated in the corroded interface with an enlarged and turbulent region.

## 4. Production of multiple value-added chemicals

The comfortable survival of human beings in contemporary society is ensured by the mass production of organic N-containing compounds. Currently, more than half of the NH_3_ produced globally is consumed in organic N-containing compounds produced industrially by thermo-catalyzed reactions under harsh conditions (150–500 °C, 20–250 bar), causing disruptions in global carbon and nitrogen cycles due to the excess emissions of CO_2_ and nitrogen oxides (NO_*x*_).^116^ Recently, with the creation of the carbon-neutral vision and the boom of CO_2_ reduction and NO_*x*_^−^ reduction, electrocatalytic C–N coupling reactions based on NO_*x*_^−^ reduction have proposed new horizons for achieving the synthesis of high-value organic N-containing compounds, which employ useless or harmful wastes including C-containing species (CO_2_, CO) and N-containing species (NO_2_^−^, NO_3_^−^).^117^ A brighter future is emerging for electrocatalytic synthesis than traditional thermo-catalytic synthesis due to the sustainability and more favourable on-site/on-demand production owing to the features of decentralisation and modularity.^118^

More intriguingly, the hidden surprises of NO_*x*_^−^ reduction extend far beyond the synthesis of high-value organic N-containing compounds. The H-type electrolyser commonly employed in the laboratory offers an uninterrupted environment for NO_*x*_^−^ reduction and also provides an opportunity for the anodic reaction to be thoroughly explored.^119^ The anodic oxygen evolution reaction (OER) commonly coupled with the NO_*x*_^−^ reduction reaction has motivated researchers to seek alternative anodic reactions including small organic molecule oxidation reaction with low energy consumption and appealing products,^120,121^ which is caused by high energy barriers and difficult collection of products in the OER. Therefore, current research advances and potential challenges will be discussed in this section on the C–N coupling reactions and alternative anodic reactions based on NO_*x*_^−^ reduction.

### 4.1 C–N coupling reactions

**Fig. 8:**
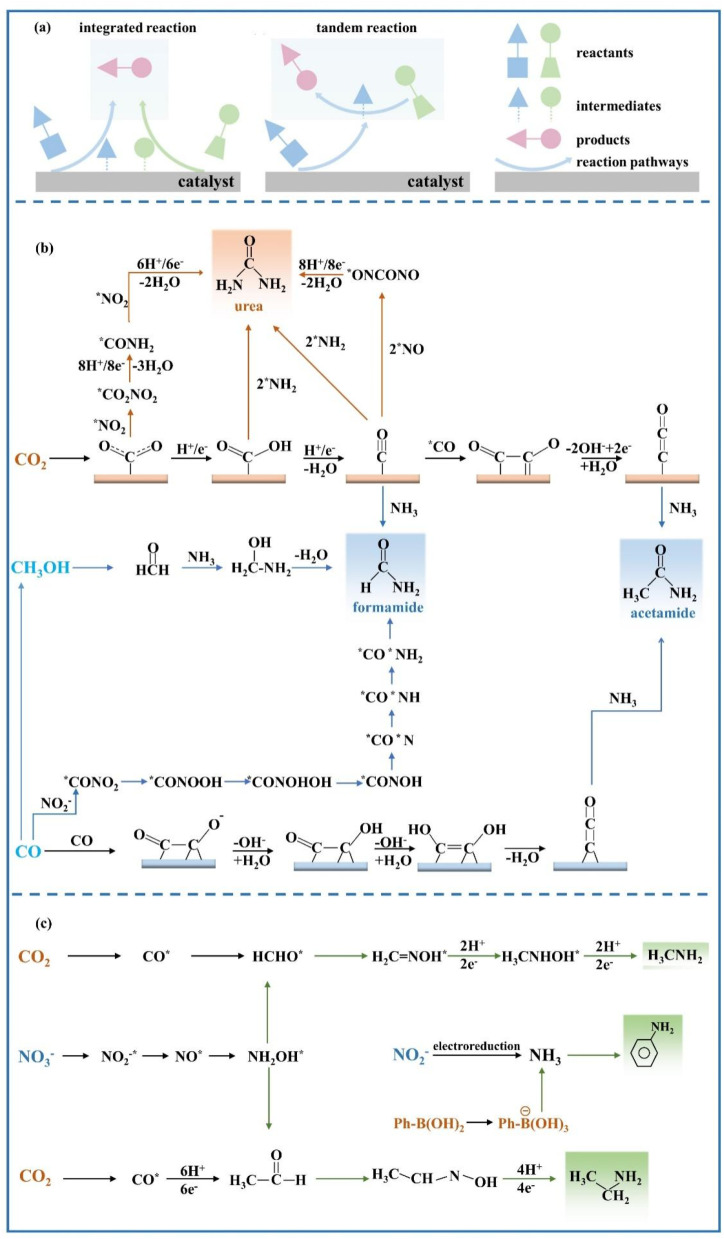
(a) Schematic diagrams of tandem reaction and integrated reaction. Reaction pathways of (b) C(=O)–N bond formation and (c) C–N bond formation.

The C–N coupling reactions based on NO_*x*_^−^ reduction can be categorized into coupling and cascade reactions, and the difference between tandem reaction and integrated reaction is illustrated in Fig. 8a. The integrated reaction is achieved by coupling key intermediates to build preset chemical bonds and generate advanced products, while tandem electrocatalytic reactions employ the *in situ* desorbed products.^122^ The integrated reaction based on CO_2_ and NO_3_^−^ reduction is commonly used for the synthesis of urea. Indeed, the NO_2_^−^ reduction process has also been confirmed to be promoted by CO_2_ on copper catalysts,^123^ which can reach a drastically enhanced NH_3_ FE of approximately 100% within a wide range of potentials. The significantly enhanced NO_2_^−^ reduction performance is due to CO_ads_ generated by CO_2_ reduction, which accelerates the deoxygenation and subsequent hydrogenation of intermediates.

The C–N coupling reaction demonstrates promising prospects for the synthesis of high value N-containing chemicals; however, a number of challenges are faced such as sluggish kinetics and low selectivity due to stubborn bonding structures of the reactants and competition of CO_2_/NO_2_^−^/NO_3_^−^ reduction and the HER.^75^ Numerous factors can exert an influence on electrocatalytic C–N coupling, including reactants, electrocatalysts and the reactors. The reactors have already been discussed in Fig. 5. The research status and the existing challenges of the C–N coupling reaction will be discussed in the part including the related reaction mechanisms and design principles of catalysts, hopefully providing a comprehensive understanding of C–N coupling reactions based on NO_*x*_^−^ reduction.

#### 4.1.1 Formation of compounds with C(=O)–N bonds

Amides are a group of compounds with a characteristic C(=O)–N unit, which can be constituted by CO and NH_*y*_ intermediates. Amides available currently based on CO/CO_2_ reduction and NO_*x*_^−^ reduction include urea, formamide, and acetamide. The possible reaction pathways of forming C(=O)–N bonds are demonstrated in Fig. 8b employing various C-containing species.

##### 4.1.1.1 Urea

Urea accounts for approximately 70% of nitrogen-containing manure in the world, contributing to the sustainable development of human society.^124^ However, it is primarily obtained through the combination of NH_3_ and CO_2_ under harsh conditions, which is adverse to alleviating fossil energy consumption and decreasing CO_2_ emissions.^125^ The synthesis of urea from CO_2_ and NO_3_^−^/NO_2_^−^ has recently been considered as a mild alternative route, but the current production efficiency is struggling to reach the standard for industrial applications.^126^ Thus, emphasis will be placed here on the formation mechanism of urea and the related studies on catalysts, in order to promote the industrial application process of electrocatalytic urea synthesis.

**Fig. 9:**
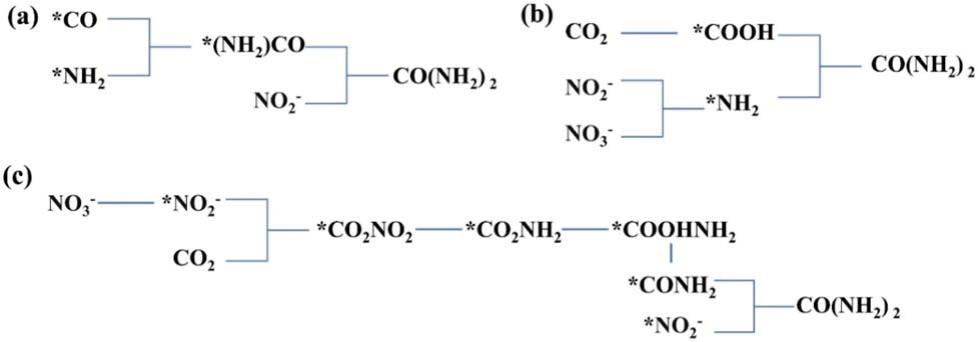
(a–c) Possible mechanisms of co-reducing electrosynthesis of urea by CO_2_ and NO_2_^−^/NO_3_^−^.

An in-depth understanding of the reaction mechanism of electrocatalytic urea synthesis is beneficial for improving the efficiency of the reaction. Numerous studies have been reported on the reaction mechanisms of electrocatalytic urea synthesis (Fig. 9), with controversies mainly existing in the key intermediates of C–N coupling. In this section, three representative mechanisms are introduced. It was proposed the formation of *(NH_2_)CO intermediates from *CO and *NH_2_ as a key step in C–N coupling through comparative experiments for the co-reduction of CO_2_ + NH_3_ and CO + NO_2_^−^.^126^ Meng *et al.* proposed that urea was generated by the coupling of *NH_2_ and *COOH intermediates in NO_2_^−^-integrated CO_2_ reduction,^127^ which was inferred from the disappearance of the signal peaks of *COOH in *in situ* diffuse reflectance infrared Fourier transform spectroscopy during the coexistence of CO_2_ and NO_2_^−^. Meanwhile, Yu *et al.* proposed that the intermediates in NO_3_^−^-integrated CO_2_ reduction are *NO_2_^−^ and *CO_2_ instead of *CO and *NH_2_.^128^ The early coupling of *NO_2_^−^ and *CO_2_ forms *CO_2_NO_2_, and subsequently, the *CO_2_NO_2_ intermediate undergoes several electron and proton transfer steps to generate *CO_2_NH_2_. The later protonation of the *CO_2_NH_2_ intermediate to *COOHNH_2_ is considered as the potential determining step (PDS) in the urea electrosynthesis process.

Despite the various controversies surrounding the current mechanisms, it is definitely evident that the adsorption configuration of CO_2_ can have a significant impact on the activity and selectivity of the urea synthesis reaction. The adsorption configurations of intermediates for the reaction can be modulated by the charged state of the catalyst surface with changes in catalyst compositions. Cu–In catalysts possessing different charge states were developed to explore the influences of different CO_2_ adsorption configurations on the activity and selectivity of the electrocatalytic synthesis of urea.^129^ The urea yield on a negatively charged Cu_97_In_3_–C catalyst with a C-bound surface was approximately thirteen times that of the positively charged Cu_30_In_70_–C catalyst possessing an O-bound surface. It was confirmed that the subsequent C–N coupling process was facilitated by the C-bound configuration (*COOH) on the catalyst surface, while the O-bond configuration (*OCHO) was a terminal blocking further non-electrochemical steps and causing inferior performance of urea formation.

With the further studies on the mechanisms of urea electrosynthesis, the focus on urea electrosynthesis has shifted to the pursuit of high FE catalysts.^130–132^ In contrast, the focus on other C–N coupling reaction organics has remained on exploring more synthetic pathways and expanding the variety of obtained N-containing organics, while less emphasis has been placed on the development of catalysts and Cu-based catalysts available commercially have usually been chosen directly. A diverse variety of catalysts have currently been developed for urea production including bimetallic catalysts, metal oxide catalysts, and monoatomic catalysts. Obviously, decent electrocatalysts with enhanced conductivity and abundant active sites are favourable to promote the simultaneous reduction of CO_2_ and NO_3_^−^/NO_2_^−^. Bimetallic electrocatalysts have exhibited promising performance in urea electrochemical synthesis, which is caused by the fact that the binding energy of the reaction intermediates can be controlled by modifying the electronic structure and composition of electrocatalysts.^133–135^ For example, Te-doped Pd nanocrystals significantly promoted the reaction between *CO and *NH_2_ in the reaction process of CO_2_ and NO_2_^−^ and inhibited the formation of N_2_ through NO_2_^−^ reduction owing to the synergistic effect between Te and Pd.^136^ The synergistic effect of bimetallic electrocatalysts was also demonstrated for specific morphological and structural regulation. Self-supported core–shell Cu@Zn nanowires, obtained by a simple electroreduction process, reached a higher urea yield rate of 7.29 μmol cm^−2^ h^−1^ and a corresponding faradaic efficiency of 9.28% compared to Zn (0.77 μmol cm^−2^ h^−1^, 1.00%) and Cu (0 μmol cm^−2^ h^−1^, 0.00%).^135^ Theoretical calculations revealed that the catalytic performance of urea electrosynthesis was enhanced by electron transfer from the Zn shell to the Cu core due to the reduction in the critical coupling energy barriers of the *CO and *NH_2_ intermediates. In comparison to metal catalysts, metal oxide catalysts were prone to introduce oxygen vacancies that acted as catalytic centres with rich electron densities.^137^ Oxygen vacancy-rich anatase TiO_2_ (Cu–TiO_2_) nanotubes could be easily obtained by low-valence Cu doping,^138^ and the high-density oxygen vacancies facilitated the selectivity of NO_*x*_ to *NH_2_ and exposure of bi-Ti^3+^ active sites.

For single-atom catalysts anchoring isolated atoms on carriers by ligands, they have aroused growing interest due to the optimal atom utilization, the explicit catalytic active sites and the absence of aggregated metal atoms.^139^ Leverett *et al.* prepared Cu–N–C single-atom catalysts for electrochemical urea synthesis and studied the effect of Cu coordination on the electrochemical reduction reactions of CO_2_ (CO_2_RR) and NO_3_^−^ (NO_3_RR).^140^ The experiments combined with theoretical calculations indicate that the Cu–N_4_ site exhibited higher activity for the CO_2_RR, while the Cu–N_4−*x*_–C_*x*_ site demonstrated a higher NH_4_^+^ yield rate in the NO_3_RR. The catalyst on the Cu–N_4_ site exhibited the best urea synthesis performance with an FE of 28% and a yield of 4.3 nmol s^−1^ cm^−2^ at −0.9 V *versus* RHE. In contrast to isolated single-atom catalysts, bonded diatomic catalysts thermodynamically and kinetically strengthen pivotal C–N coupling due to the presence of effective sites for coordinated adsorption and coactivation of carbon and nitrogen sources. The bonded diatomic Fe–Ni catalyst demonstrated excellent performance, reaching a high urea yield of 20.2 mmol h^−1^ g^−1^ with FE of 17.8%,^141^ which was up to an order of magnitude higher than those of single-atom and isolated diatomic electrocatalysts. Such excellent performance is mainly due to two factors: (1) the simultaneous introduction of Fe and Ni sites overcomes the restriction of unilateral selective adsorption and activation of carbon or nitrogen reactants. (2) The bridge sites of Fe–Ni pairs boost the C–N coupling process thermodynamically and kinetically, and the bridged configuration inhibits the HER effectively.

The metal-based catalysts mentioned above have exhibited promising performance; nonetheless, metal-based catalysts are still hampered by high cost,^142^ destabilization under adverse operating conditions^143^ and vulnerability to small molecule toxicity.^144^ Carbon-based metal-free electrocatalysts have shown brilliant application prospects in the electrocatalytic synthesis of urea due to abundant sources, competitive cost and superior stability. The doping of heteroatoms such as N, B and F has been widely employed for optimizing the electrocatalytic properties of carbon materials for urea synthesis due to new surface charge distribution induced by doping. The HER activity can be suppressed in carbon materials doped with F, facilitating enhanced urea synthesis activity. The high urea yield rate of carbon nanotubes with a fluorine-rich surface (F-CNT) could reach up to 6.36 mmol g_cat_^−1^ h^−1^, which has been confirmed to be caused by more favorable *COOH generation and *NH_2_ formation processes on the F-CNT.^143^ The abundant nitrogen-containing active intermediates are also more conducive for urea synthesis. The remarkable urea yield rate of 610.6 mg h^−1^ g_cat_^−1^ was exhibited in porous N-doped carbon obtained by pyrolysis of the coordination polymer, which even exceeded those of some noble metal-based catalysts.^145^

The synthesis of urea by simultaneous electrochemical reduction of CO_2_ and NO_2_^−^/NO_3_^−^ has gained growing attention, especially when the coupled CO_2_RR is of high significance for achieving carbon neutrality. However, the synthesis mechanisms are full of arguments. In regard to studies on catalysts, numerous experiments in the design of high-performance catalysts have confirmed that it is imperative to attach importance to the coactivation and reaction of reactants, as well as the construction of efficient sites conducive to C–N coupling by optimizing the adsorption of intermediate components. Nevertheless, the Faraday efficiencies of current catalysts for urea synthesis are generally lower than 70%,^146^ which are far from the actual requirements. Furthermore, the precise regulation of the interfacial microenvironment should not be ignored for comprehensively improving the electrocatalytic performance, since efficient and stable three-phase interfaces are required to supply reactants and accelerate mass transfer.^147^ Three optimization strategies can be employed for controlling the microenvironment, including adjusting the hydrophobicity of electrocatalysts, improving proton supply in the electrolyte and regulating experimental conditions in the electrolyzer.

##### 4.1.1.2 Organic amides

Amides including formamide and acetamide are of significant commercial value, and are widely employed in polymer manufacture and biological compounds. In view of the energy crisis and environmental pollution aggravated by the current industrial synthesis of amides, electrosynthesis routes have been developed employing CO_2_ and NH_3_. The combination of NH_3_ in the liquid phase and CO_2_ in the gas phase has been proven to successfully synthesize formamides and acetamides over non-homogeneous Cu catalysts,^148^ the detailed mechanisms are shown in Fig. 8b. However, the FEs obtained are considerably low, not even exceeding 1% at the highest. In addition, the electrolysis process will be disrupted by the undesired carbonate formation at the electrode–electrolyte interface, owing to the inevitable reaction of OH^−^ ions with CO_2_ under alkaline conditions.

In order to solve such dilemma, CO reduction is a promising strategy to replace direct CO_2_ reduction, which can yield CO from electrochemical CO_2_ reduction under non-alkaline conditions. The higher selectivity for acetic acid in CO reduction has been demonstrated compared to CO_2_ reduction on the Cu catalyst surface, implying an easy reaction between the ketene intermediates in the CO reduction process with the nucleophilic agent NH_3_. The FE of generating acetamide can reach 40% at −0.68 V *vs.* RHE on the Cu nanoparticle catalyst, when the molar ratio of CO to NH_3_ is up to 2 : 1.^149^ The synthesis mechanism and competitive reactions for acetamide formation are shown in Fig. 8b. Under the conditions of high pH and less negative potential, CO reduction is more biased towards the generation of the C=C=O intermediate compared with the generation of the C=COH intermediate for producing ethylene and ethanol. Although the formation of ethylene and ethanol is inhibited at high NH_3_ concentration, the formation of acetamide is also faced with competition for forming acetate due to OH^−^ on the surface of the catalyst.

The N-containing nucleophilic reagent in the above C–N coupling pathways directly employs NH_3_. With the flourishing of studies on NO_2_^−^ reduction to NH_3_ due to the low dissociation energy of the N=O bond (204 kJ mol^−1^), a credible route is provided for green NH_3_ production with renewable electricity. Electrocatalytic coupling of NO_2_^−^ with CO has been confirmed to be an alternative avenue to achieve formamide synthesis. The reaction pathway is shown in Fig. 8b for electrocatalytic coupling of NO_2_^−^ with CO obtained by theoretical calculation, and the key challenge for reaching decent formamide selectivity is the construction of highly active and stable catalysts for enhancing CO and NO_2_^−^ activity reduction and promoting C–N coupling. Ru atoms dispersed on Cu nanoclusters (Ru–Cu) have been developed to achieve decent formamide FE of 45.65% with a yield of 2483.77 μg h^−1^ mg_cat_^−1^ at −0.5 V *vs.* RHE.^150^ The design of dual active sites in Ru–Cu catalysts could achieve synergistic catalysis for C and N activation, which can significantly improve the C–N coupling efficiency compared to monometallic catalysts. The adsorption and subsequent hydrogenation process of NO_2_^−^ could be promoted by Ru atoms, while the dissociation adsorption of CO could be accelerated by adjacent Cu sites. Therefore, the decent activity and selectivity for formamide formation can be reached by the synergistic catalysis in Ru–Cu catalysts.

The electrocatalytic C–N coupling system developed currently is generally employed under aqueous conditions, and the further enhancement of C–N coupling efficiency is limited by severe HER competition due to the low-soluble CO in the aqueous solution. The extremely water-soluble methanol can be obtained from CO conversion, which can be an attractive alternative C-containing species for C–N coupling reactions. The combination of methanol and NH_3_ has provided an interesting process to produce formamide under ambient conditions, which utilizes the nucleophilic attack of NH_3_ on a formaldehyde-like intermediate from methanol electrooxidation. The most likely reaction pathway is illustrated in Fig. 8c. The C–N bond formation in formamide is caused by a nucleophilic attack process, where the positively charged C in *CH_2_O is attacked by the electronegative N atom in NH_3_. The conversion of methanol and NH_3_ to formamide can reach a selectivity of 74.26% and FE of 40.39% on PtO_2_ at 100 mA cm^−2^.^151^ The decent formamide production efficiency was due to the moderate affinity of the reaction intermediate on PtO_2_. However, the large-scale production of formamides based on methanol and NH_3_ is faced with the limitation of poor mass transfer at high current density exceeding 100 mA cm^−2^. Compared with the easy dissolution of precious metal Pt under large oxidation current density, a boron-doped diamond (BDD) electrode is highly promising for large-scale formamide electrosynthesis due to the outstanding stability on the most corrosive electrolytes with a wide potential window.^152^ The BDD electrode exhibits high durability after a continuous 20-cycle test on a laboratory scale, which also shows decent FE of 33.5% and output of 36.9 g h^−1^ at 264 A in the pilot plant test.

The combination of methanol and ammonia has provided a favorable idea for the electrochemical synthesis of formamide, and this strategy has been proven to be extensible for the synthesis of other organic N compounds. More intriguingly, acetamide and propenamide can be obtained by lengthening the C chain of C-containing species, and formyl methylamine can be obtained by replacing the N source (CH_3_NH_2_).

#### 4.1.2 Formation of compounds with C–N bonds

Numerous co-reduction experiments with CO_2_ and NO_2_^−^/NO_3_^−^ have successfully generated urea, which demonstrates the potential of producing organonitrogen compounds. Nevertheless, the current scope of reaction products requires further broadening to produce more high-value chemicals, such as alkylamines. Wang *et al.* has made breakthroughs in the co-reduction of CO_2_ and NO_3_^−^ to produce alkylamines, including methylamine^29^ and ethylamine,^153^ involving more advanced catalysts with novel C–N coupling mechanisms.

**Fig. 10:**
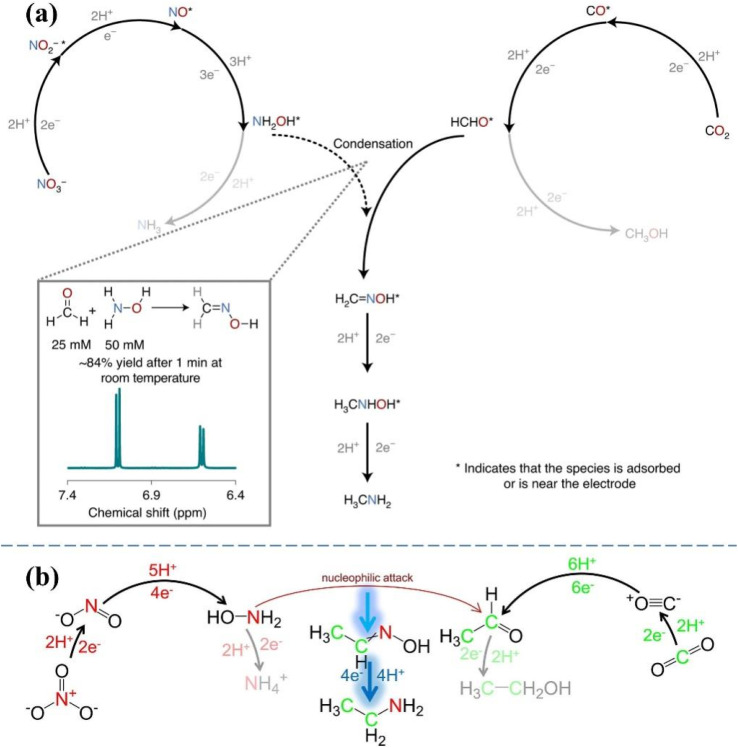
(a) The proposed reaction pathways to form methylamine. Reproduced with permission.^29^ Copyright 2021, Springer Nature. (b) Reaction pathways for obtaining ethylamine. Reproduced with permission.^155^ Copyright 2022, Elsevier.

Methylamine is the simplest alkylamine and is employed as the major commercial chemical intermediate in pesticide production, solvent fabrication and water treatment.^154^ For industrial production, methylamine is currently obtained from methanol (CH_3_OH) and NH_3_ under high-temperature high-pressure conditions. CoPc-NH_2_/CNT was developed as a working electrode for driving the co-reduction process of CO_2_ and NO_3_^−^ to methylamine involving the transfer of 14 electrons and 15 protons.^29^ The total FE of the co-reduction process reaches 13%, with no performance degradation after at least 16 hours of uninterrupted operation. More significantly, the intermediates (NH_2_OH and HCHO) involved in the key C–N coupling step are confirmed; thus, eight consecutive reaction steps regarding the formation of methylamine are proposed. As shown in Fig. 10a, formaldoxime is generated by spontaneous condensation through NH_2_OH derived from NO_3_^−^ with HCHO obtained from CO_2_, and then formaldoxime is reduced to form methylamine.

The synthesis of ethylamine is obviously more challenging than that of methylamine, which is caused by the process of transferring 20 electrons and 21 protons in total. The cascade electrocatalytic synthesis of ethylamine from CO_2_ and NO_3_^−^ is achieved by the catalysis of oxide-derived Cu nanoparticles.^153^ In addition, the related synthesis mechanism is proposed. The mechanism of ethylamine synthesis displayed in Fig. 10b is similar to that of methylamine, and the critical C–N coupling step is the condensation of hydroxylamine (NH_2_OH) with aldehyde (CH_3_CHO) to form acetaldoxime. However, the FE of ethylamine production by this process is as low as 0.3%. The poor yield of ethylamine is primarily attributed to the following factors: (1) it is adversely affected by the competition between the rapid side reactions of NH_2_OH to NH_4_^+^ and CH_3_CHO to CH_3_CH_2_OH. (2) The reduction rate of acetaldoxime is significantly slower than that of the CO_2_RR, NH_3_RR and HER. (3) The selectivity of the CO_2_ to CH_3_CHO reduction pathway on Cu-based catalysts is inferior.

While significant breakthroughs have been made in the synthesis of methylamine and ethylamine, it is hard to implement them immediately in industry. Apparently, the electrocatalytic synthesis of propylamines or alkylamines with more C atoms will also be more arduous. The route for generating arylamine has recently been explored by coupling arylboronic acid with NH_3_ produced from NO_2_^−^ reduction. Intriguingly, the synthesis of arylamine was carried out at pulsed potentials,^156^ which was owing to the consideration that metal electrodes were easily reduced to zero-valent metals during NO_2_^−^ reduction and deactivated due to excessive oxidation in the C–N coupling process. Therefore, low-coordinated Cu nano-coral was employed under pulse potential for efficient NO_2_^−^ reduction and C–N coupling, which experienced an alternating transition from the zero-valent state to the divalent state.

#### 4.1.3 Other possible products

Expanding the possible range of C–N bonds that can be formed has been a hot topic to obtain more abundant and high value-added organic nitrogen compounds.^150^ It is evident from the synthetic routes discussed above that the majority of the synthetic pathways proposed have been essentially based on nucleophilic attack of N-containing species on C-containing species, which hints at the opportunity of obtaining more abundant N-containing organics by altering the N and C sources. For example, it has been proven to be possible to form alanine by replacing the C source with pyruvic acid,^157^ which is coupled with NH_2_OH intermediates during NO_3_^−^ reduction.

The environmental pollutant CO could be a promising C source substituting CO_2_, which facilitates environmental restoration and averts the disruption of the electrolysis process due to undesirable carbonate formation from the inevitable reaction of OH^−^ with CO_2_ at the electrode–electrolyte interface. The possible C and N sources and the corresponding theoretical products are depicted in Table 2, presumably contributing to the broadening of the organonitrogen product scope to obtain more high-value products.

**Table 2:** Possible organonitrogen products with corresponding C sources and N sources

C source	N source	Organonitrogen compound
CO	NH_3_	Acetamide
CO	CH_3_NH_2_	*N*-Methylacetamide
CO	C_2_H_5_NH_2_	*N*-Ethylacetamide
CO	(CH_3_)_2_NH	*N*,*N*-Dimethylacetamide

### 4.2 Valuable anode reactions coupled with NO_*x*_^−^ reduction

The traditional NO_*x*_^−^ reduction reaction is usually coupled with the OER, which consumes up to 90% of the input energy and produces low-value O_2_.^158^ The OER with high energy consumption results in twice the production cost of the electrocatalytic NH_3_ production compared to the conventional Haber–Bosch route for producing NH_3_ with the same quality.^2^ Currently, appeals to develop alternative oxidation reactions are progressively increasing for enhancing energy utilization efficiency and obtaining value-added chemicals. The development of anodic oxidation reactions employing inexpensive or hazardous reactants has received considerable attention. Electrooxidation of N_2_ to HNO_3_ is a promising alternative reaction, which can both generate high-value HNO_3_ and provide reactants for NO_*x*_^−^ reduction.^119^ In spite of the inferior FE of only 1.23% for N_2_ oxidation on Pt foil,^159^ this strategy employing anodic N_2_ oxidation has provided a promising route for obtaining HNO_3_ and NH_3_ at distributed sources.

**Fig. 11:**
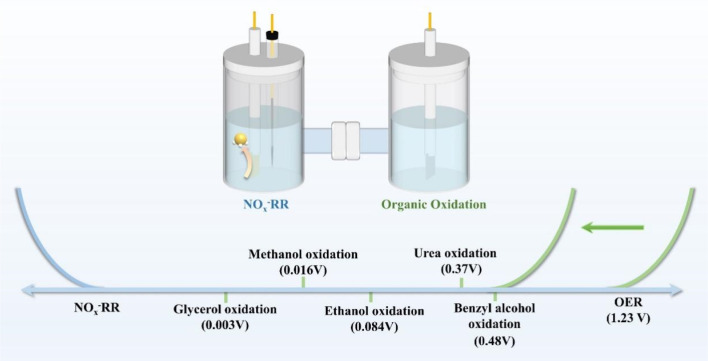
Thermodynamic equilibrium potentials of several oxidation reactions.

In addition, the immense interest in electrooxidation reactions of biomass and related derivatives has been driven not only by lower theoretical potentials than those of the OER (Fig. 11) but also by the sustainable nature for accelerating the creation of a carbon-neutral society.^160^ Biomass and its derivatives have been considered as renewable carbon-neutral resources including 5-hydroxymethylfurfural, glycerol and benzyl alcohol with abundant proton content.^161–165^ Commercial Ni foam was usually employed as an anode in earlier studies for initially verifying the feasibility of replacing the anodic reaction. With the deeper cognition on electrocatalytic reconfiguration, excellent bifunctional catalysts can be obtained by adjusting the reconfiguration direction of catalysts in different reaction processes.^166^ Decent bifunctional NiCu based catalysts have been developed for NO_3_^−^ reduction and glycerol oxidation reactions, which can be reconstructed under different operating conditions.^167^ Under the cathodic reduction environment, the materials were transformed into amorphous Ni(OH)_2_ coupled Cu nanoparticles. The NO_3_^−^ reduction performance of the material was enhanced by the synergistic effect of Cu and Ni(OH)_2_. Meanwhile, composites including NiOOH and CuO with rich Cu vacancies were obtained by the reconstruction of NiCu based catalysts during the glycerol oxidation process. The glycerol oxidation process was promoted by the increased exposure of active NiOOH species due to the leaching of Cu in Cu vacancy-rich CuO. Compared to the traditional electrolyzer coupled with the OER, the electrolyzer coupled with the GOR exhibited an incredible overpotential reduction of 285 mV at a current density of 100 mA cm^−2^.

Recently, some efforts have been focussed on further improving the economic efficiency of electrochemical systems coupled with NO_3_^−^ reduction and glycerol oxidation. A CO_2_ capture strategy has been explored for upgrading products,^168^ which can convert anodic formate to potassium diformate and cathodic NH_3_ to NH_4_HCO_3_. This strategy is conducive to product separation holding promising prospects for industrial applications.

The strategy of replacing the OER with biomass electrooxidation has provided an innovative insight for reducing electricity consumption and enhancing product value. However, biomass electrooxidation also faces the challenges of higher cost and higher requirements for membrane, which typically drives current density below 200 mA cm^−2^ under potential exceeding 1.23 V.^169–171^ The undesirable overpotential in the biomass electrooxidation process may be attributed to the high energy required for destroying C–H and O–H bonds in biomass.^172,173^ Compared to the C–H and O–H bonds in alcohols or aldehydes, more easily dissociated hydrogen atoms are manifested in the more reactive enol structure.^174^ Consequently, substances including ascorbic acid with a highly active enol structure can be considered as an anode additive to accelerate NH_3_ production in future efforts.

## 5. Energy conversion and storage systems

Metal–NO_2_^−^/NO_3_^−^ batteries have received growing attention as a paradigm of simultaneous NH_3_ production and energy output.^175–178^ The anodes currently employed in metal–NO_2_^−^/NO_3_^−^ batteries are dominated by Zn anodes, which are especially appealing in the treatment of industrial wastes containing NO_2_^−^/NO_3_^−^ due to their low cost, easy recycling and high stability in alkaline solutions.^179^ The working mechanism, performance, hurdles and opportunities of Zn–NO_2_^−^/NO_3_^−^ batteries are primarily discussed in this section.

### 5.1 Metal–NO_3_^−^ batteries

**Fig. 12:**
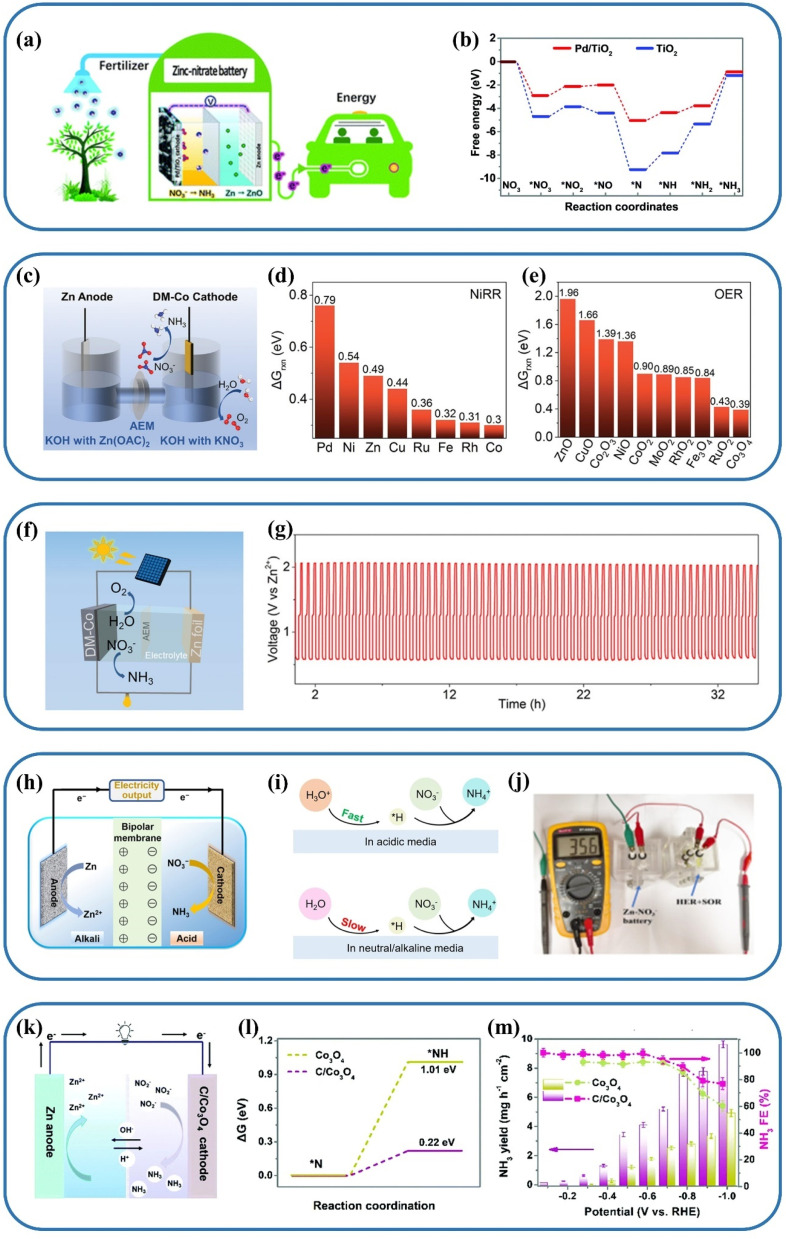
(a) Schematic representation of the Zn–NO_3_^−^ battery, (b) the calculated intermediate adsorption energies on Pd/TiO_2_. Reproduced with permission.^21^ Copyright 2021, Royal Society of Chemistry. (c) Schematic diagram of a rechargeable Zn–NO_3_^−^ battery. Catalyst screening studies of (d) the NO_3_RR and (e) OER. (f) Photovoltaic driven Zn–NO_3_^−^ battery. (g) Galvanostatic discharge–charge cycling curves of a Zn–NO_3_^−^ battery assembled from DM-Co. Reproduced with permission.^180^ Copyright 2022, Wiley-VCH. (h) Schematic diagram of an alkaline–acidic hybrid Zn–NO_3_^−^ battery. (i) Pathways for active hydrogen production in the NO_3_^−^RR process under different conditions. (j) Environmental sulfur recovery powered by a hybrid Zn–NO_3_^−^ battery. Reproduced with permission.^181^ Copyright 2023, Springer Nature. (k) Schematic diagram of a Zn–NO_2_^−^ battery. (l) The energy barriers of C/Co_3_O_4_ and Co_3_O_4_ for the rate-determining step in the NO_2_RR. (m) NO_2_RR performance of C/Co_3_O_4_ at different potentials. Reproduced with permission.^23^ Copyright 2022, Royal Society of Chemistry.

In 2021, the viability of galvanic metal–NO_3_^−^ batteries (Fig. 12a) was evidenced by Zhi's group for the first time, shedding renewed light on the field of sustainable NH_3_ generation and zinc-based batteries.^21^ A Pd-doped TiO_2_ nanoarray was employed as the cathode in this experiment, which exhibited impressive NO_3_^−^ reduction activity due to attenuated intermediate adsorption (Fig. 12b) with introducing Pd. The electrochemical reactions of the discharge process in this Zn–NO_3_^−^ cell are as follows:Cathode reaction: NO_3_^−^ + 7H_2_O + 8e^−^ → NH_4_OH + 9OH^−^Anode reaction: 4Zn + 8OH^−^ → 4ZnO + 4H_2_O + 8e^−^Overall reaction: 4Zn + NO_3_^−^ + 3H_2_O → 4ZnO + NH_4_OH + OH^−^

However, the rechargeability of Zn–NO_3_^−^ batteries has not been sufficiently investigated in the Zn–NO_3_^−^ battery with Pd-doped TiO_2_ as the cathode. Lin *et al.* have developed rechargeable Zn–NO_3_^−^ batteries (Fig. 12c), inspired by soybean which could exploit nitrogen and generate oxygen simultaneously. Bifunctional DM-Co catalysts were designed for the NO_3_RR and OER after theoretical pre-screening (Fig. 12d and e), which reveals the great potential of Co-based catalysts.^180^ The aqueous rechargeable Zn–NO_3_^−^ battery constructed with DM-Co as the cathode achieves a high power density of over 25 mW cm^−2^, which is much higher than that of the Zn–NO_3_^−^ battery with a Pd-doped TiO_2_ cathode (0.87 mW cm^−2^). In addition, the successive discharge–charge cycle curves illustrated in Fig. 12g reveal the excellent robustness of the rechargeable Zn–NO_3_^−^ battery, presenting 76 cycles at a low potential of 2.1 V. The discharge process in this rechargeable Zn–NO_3_^−^ cell is analogous to that of the Zn–NO_3_^−^ cell with a Pd-doped TiO_2_ cathode.

The following reactions take place when the aqueous Zn–NO_3_^−^ battery is charged:Cathode reaction: 4OH^−^ → 2H_2_O + O_2_ + 4e^−^Anode reaction: Zn(OH)^2^_4_^−^ → Zn^2+^ + 4OH^−^Zn^2+^ → Zn − 2e^−^Overall reaction: 2Zn(OH)^2^_4_^−^ → 2Zn + 2H_2_O + 4OH^−^ + O_2_

The total battery reaction of the rechargeable Zn–NO_3_^−^ cell can be described as follows:NO_3_^−^ + 3H_2_O → NH_4_^+^ + 2OH^−^ + 2O_2_

It is worth mentioning that the Zn–NO_3_^−^ battery system driven by a photovoltaic cell (Fig. 12f) has also been attempted. When the Zn–NO_3_^−^ battery is charged, the electrical energy is stored in the chemical bonds of the Zn anode, which is converted from the solar energy absorbed by the photovoltaic cell. The optimal NO_3_RR FE of 95% and solar-to-NH_3_ efficiency of 19.5% are obtained in the Zn–NO_3_^−^ cell system driven by a photovoltaic cell. The cells described above are operated in alkaline environments; however, corrosion issues of equipment and limitations on the type of battery components are unavoidable under extreme pH conditions.^182^ A Zn–NO_3_^−^ cell with a Co_2_AlO_4_ cathode has also recently been constructed in a neutral environment,^40^ which is also beneficial for simulating the actual textile wastewater environment. The introduction of Al ions improves CO_3_O_4_ with poor ammonia production performance in a neutral environment, which achieved optimal adsorption of NO_3_^−^ on Co sites by reducing the electron cloud density on the Co surface. The power density of 3.43 mW cm^−2^ offered by the Zn–NO_3_^−^ battery was higher than that of the Zn–NO_3_^−^ battery reported for the first time. However, the output voltages are limited in current Zn–NO_3_^−^ batteries equipped with NO_3_^−^ reduction under neutral/alkaline conditions, which indicates more challenges on the conditions of the cathodic part severely affecting the power density and NH_3_ yield of the related battery. Recently, an alkaline–acidic hybrid Zn–NO_3_^−^ battery (Fig. 12h) is developed to exhibit higher output power density, due to enhanced NO_3_^−^ conversion rate and more energy-efficient NH_3_ generation with abundant protons provided in an acidic environment (Fig. 12i). FePc/TiO_2_ is developed as a stable and active electrocatalyst for energy-efficient acid NO_3_^−^ reduction with an impressive NH_3_ yield rate of 17.4 mg h^−1^ cm^−2^ and NH_3_ FE of 90.6%.^181^ The developed alkaline–acid hybrid Zn–NO_3_^−^ battery based on the FePc/TiO_2_ cathode shows high open-circuit voltage up to 1.99 V with high power density of 91.4 mW cm^−2^, which can be applied for efficient environmental sulfur recovery by driving the electrolyzer composed of cathodic HER and anodic sulfur oxidation reaction with the current density of 35.6 mA cm^−2^ (Fig. 12j).

Some progress has been made in terms of rechargeability studies of Zn–NO_3_^−^ batteries and the design of cathodes under different conditions. However, the performance of the cells requires further enhancement and the electrolyte environment in the cells should be brought closer to that of real wastewater. Notably, the potential value of the NO_3_RR under basic conditions is 0.6 V *vs.* the RHE, which is higher than the 0.4 V of the oxygen reduction reaction (ORR) O_2_ + 2H_2_O + 4e^−^ → 4OH^−^.^183^ Consequently, NO_3_^−^-based batteries potentially produce a higher voltage output than metal–air batteries. The performances of Zn–NO_3_^−^ batteries and Zn–air batteries as reported in current literature are presented in Table 3. It is clear that a significant discrepancy can be found between the performance of the Zn–NO_3_^−^ batteries and the Zn–air batteries; reducing the discrepancy can be achieved by seeking decent-efficiency and high-selectivity catalysts and developing more Zn–NO_3_^−^ batteries with an abundant electrolyte environment.

**Table 3:** Comparison of the performance between Zn–NO_2_^−^/NO_3_^−^ batteries and various Zn-based batteries

Batteries	Catalyst	OCV (V)	FE (%)	NH_3_ yield (mg h^−1^ cm^−2^)	Power density (mW cm^−2^)	Ref.
Zn–NO_3_^−^	Pd/TiO_2_	0.81	81.3	0.54	0.87	21
Zn–NO_3_^−^	ZnCo_2_O_4_	0.6	98.33	1.55	4.62	22
Zn–NO_3_^−^	Fe/Ni_2_P	1.22	85	4.17	3.25	176
Zn–NO_3_^−^	Co_2_AlO_4_	1.862	92.6	0.75	3.43	40
Zn–NO_3_^−^	NiCo_2_O_4_/CC	1.30	96.1	0.82	3.94	175
Zn–NO_3_^−^	CeO_2−*x*_@NC	1.45	96.09	2.46	3.44	184
Zn–NO_3_^−^	DM-Co	0.62	91	2.04	25	180
Zn–NO_3_^−^	CuNi NPs/CF	0.94	97.03	94.57	70.7	185
Zn–NO_3_^−^	FePc/TiO_2_	1.99	88.2	12.3	FePc/TiO_2_	181
Zn–NO_2_^−^	TiO_2−*x*_	0.6	91.1	12.230	2.38	177
Zn–NO_2_^−^	C/Co_3_O_4_	1.589	95.1	0.802	6.03	23
Hydrazine-nitrate	Bimetallic RuCo	—	—	6.64	12	178
Zn–N_2_	CoPi/NPCS	∼1.4	16.35	0.0147	0.49	186
Zn–N_2_	OV-Ti_2_O_3_	—	19.29	0.03724	1.02	187
Zn–N_2_	VN@NSC-900	∼0.55	—	0.000172	0.01642	18
Zn–N_2_	CoPi/HSNPC	∼1	24.42	—	0.31	188
Zn–N_2_	NbS_2_	0.5	10.12	0.03758	0.31	189
Zn–N_2_	Fe_1.0_HTNs	—	—	0.00014	0.028	190
Zn–NO	MoS_2_	2.03	85.0	0.4118	1.04	191
Zn–NO	CoS_1−*x*_	1.83	53.62	1.49	2.06	192
Zn–NO	Bi@C	2.08	93	0.36	2.35	193
Zn–O_2_	S–FeCo_3_P/NPSG	—	—	—	38	194
Zn–O_2_	Fe@Co-NMC	—	—	—	98.7	195
Zn–O_2_	AP-CONPs/NF	1.37	—	—	89.1	196

### 5.2 Metal–NO_2_^−^ batteries

The cathode conditions of Zn–NO_2_^−^ batteries (Fig. 12k) developed currently are an alkaline environment and neutral environment,^177^ and the cathode reaction in the alkaline environment is identical to that in the neutral environment. Carbon-doped Co_3_O_4_ nanotubes have been developed as cathodic catalysts under neutral conditions for the assembly of novel Zn–NO_2_^−^ batteries.^23^ During the NO_2_^−^ reduction process, the energy barrier of *N hydrogenation in C/Co_3_O_4_ (Fig. 12l) is significantly reduced due to accelerated charge transfer caused by the C dopant inducing a local electric field. Carbon-doped Co_3_O_4_ possessed decent catalytic activity (Fig. 12m) for NO_2_^−^ reduction, achieving a high FE approaching 100% for ammonia production within a wide range (−0.1 V to −0.6 V *vs.* RHE). The assembled Zn–NO_2_^−^ battery demonstrated a power density of 6.03 mW cm^−2^ and a FE of 95.1% for NH_3_ production.

The electrochemical reactions in the Zn–NO_2_^−^ battery are presented as follows:Cathode reaction: NO_2_^−^ + 6H_2_O + 6e^−^ → NH_4_OH + 7OH^−^Anode reaction: 3Zn + 6OH^−^ → 3ZnO + 3H_2_O + 6e^−^Overall reaction: 3Zn + NO_2_^−^ + 3H_2_O → 3ZnO + NH_4_OH + OH^−^

Some advances have been made in Zn–NO_2_^−^ batteries with cathodes under neutral and alkaline conditions. Indeed, little effort has been devoted to catalysts in acidic environments, while the theoretical voltage (2.146 V) of the Zn–NO_2_^−^ cell in the acidic environment is higher than those in alkaline (1.089 V) and neutral environments (1.589 V). Furthermore, a bottleneck currently exists in the development of rechargeable Zn–NO_2_^−^ batteries, which may be due to the challenge in avoiding the OER coinciding with the conversion of nitrite to nitrate.

### 5.3 Hurdles and opportunities on batteries

The comparative performances of the currently developed Zn–NO_2_^−^ batteries, Zn–NO_3_^−^ batteries and Zn–gas batteries are exhibited in Table 3, thus providing a better understanding of the development status of Zn–NO_2_^−^ batteries and Zn–NO_3_^−^ batteries. It is evident that Zn–NO_*x*_^−^ batteries are much more promising than Zn–N_2_ for simultaneous NH_3_ generation and electricity output. However, the insufficiently desirable state of current Zn–NO_*x*_^−^ batteries must also be confirmed. Strategies to improve the performance of Zn–NO_*x*_^−^ batteries cannot be limited in designing more advanced cathode catalysts with reduced HER competition.^197^ As shown in Table 3, despite facing fierce competition from the HER, the FEs for Zn–NO_*x*_^−^ batteries range from 81.3% to 98.3%, which suggests that the HER at most leads to 1.7–18.7% loss in FE. In view of the higher theoretical voltage of the Zn–NO_*x*_^−^ cell and more abundant proton supply in an acidic environment, it is essential to develop a Zn–NO_*x*_^−^ cell with acidic cathodic NO_*x*_^−^ reduction and enhance the corrosion resistance of the corresponding electrodes for effectively increasing the open circuit voltage and power density.

Moreover, pure metallic Zn electrodes are widely employed in rechargeable battery systems, including zinc–air, silver–zinc and zinc–nickel batteries;^198–200^ however, many adverse side reactions exist between Zn electrodes and the electrolyte during the reaction process, such as electrode passivation, zinc dendrite growth and electrode distortion.^201–203^ In particular, the growth of zinc dendrites is caused by the inhomogeneous deposition of Zn during the charging process,^204^ which can cause drastic degradation of the coulomb efficiencies and capacities of the batteries. More seriously, the two electrodes of the battery will come into contact when the dendrites pierce the membrane of the battery, leading to internal short-circuiting and termination of the battery. Various approaches have been proposed to combat zinc dendrites, including electrolyte optimization,^205^ electrode surface modification^206^ and electrode structure design;^207^ nevertheless, comprehensive criteria for evaluating the state of metal anodes in aqueous metal–NO_2_^−^/NO_3_^−^ batteries are currently lacking. Thus, the behavior of metal anodes in aqueous metal–NO_2_^−^/NO_3_^−^ batteries could be considered as a priority for future studies.

Apart from the influence of Zn, the robustness of metal–NO_2_^−^/NO_3_^−^ batteries is also affected by the consumption of NO_2_^−^/NO_3_^−^. To overcome this issue, a continuously stirring flow system can be introduced with steady NO_2_^−^/NO_3_^−^ concentration and stable current density. Future efforts can be attempted in the design and construction of highly efficient and selective electrocatalysts applied in a flow metal–NO_2_^−^/NO_3_^−^ battery system.

**Fig. 13:**
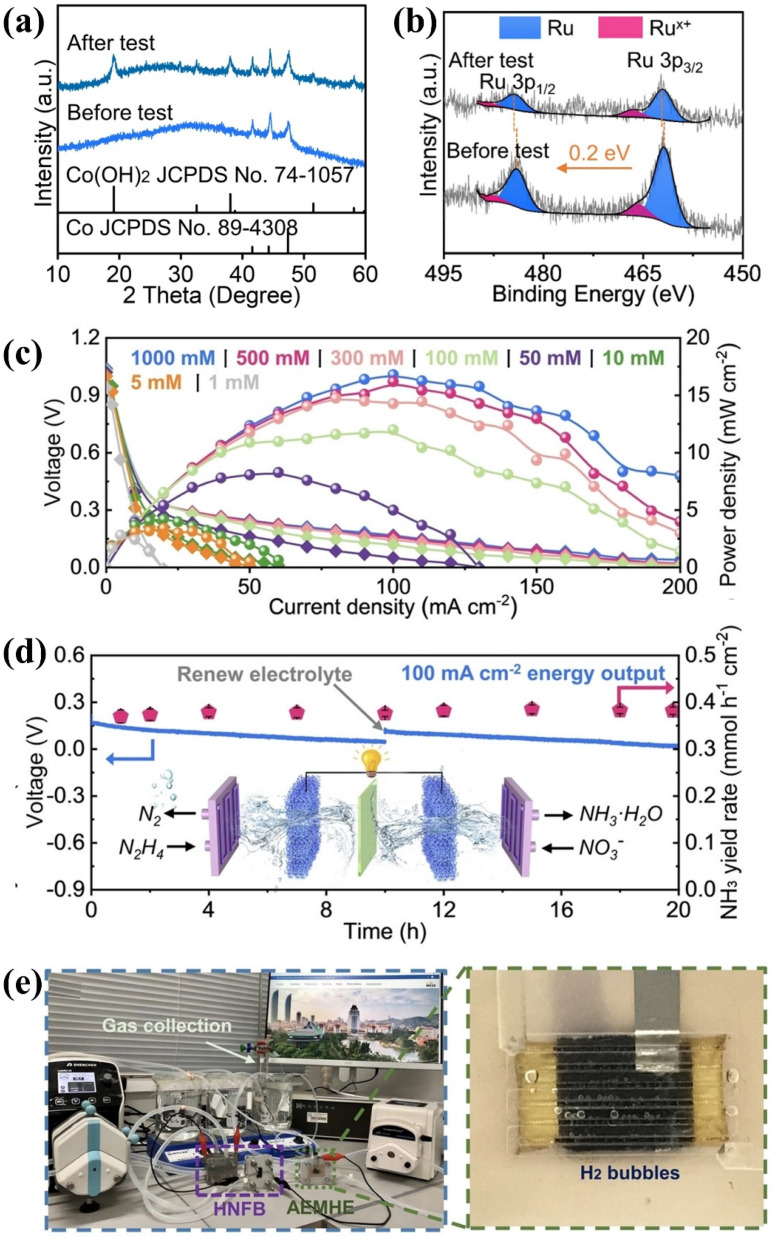
(a) XRD pattern and (b) Ru 3p spectra of RuCo catalysts before and after testing. (c) Power density curves and (d) stability test of the N_2_H_4_–NO_3_^−^ flow battery. (e) Digital photograph of the tandem N_2_H_4_–NO_3_^−^ flow battery and hydrogen production electrolyzer. Reproduced with permission.^178^ Copyright 2023, Wiley-VCH.

In addition to enhancing the metal–NO_2_^−^/NO_3_^−^ battery performance by optimizing electrode catalysts and metal anodes, some attempts have recently been made to replace the Zn oxidation reaction with other oxidation reactions including hydrazine (N_2_H_4_) oxidation. Theoretically, a battery can be composed of anodic N_2_H_4_ oxidation to N_2_ and cathodic NO_3_^−^ reduction to NH_3_. In this battery, a high theoretical discharge voltage can be generated up to 1.04 V (NO_3_^−^ + 2N_2_H_4_ → NH_3_ + 2N_2_ + 2H_2_O + OH^−^),^208,209^ accompanied by sewage purification and NH_3_ production. Currently, in view of the importance of electrolyte renewal, a novel N_2_H_4_–NO_3_^−^ flow battery has been developed, which employs RuCo precatalysts as electrodes for accelerating both the N_2_H_4_ oxidation and NO_3_^−^ reduction.^178^ The RuCo precatalysts have been confirmed to be reconstructed into Ru/Co(OH)_2_ heterostructures during the electrocatalysis (Fig. 13a). The positive shift of 0.2 eV could be observed in the Ru 3p XPS peaks of the precatalyst after the test (Fig. 13b), suggesting the formation of electron-deficient Ru sites and strong interfacial interactions between Ru and Co(OH)_2_. The battery performances influenced by wastewater concentration are depicted in Fig. 13c. The discharge power density is gradually enhanced from 2.8 to 16.8 mW cm^−2^ with the increase of concentration from 1 to 1000 mM. Fig. 13d illustrates the stability test of the N_2_H_4_–NO_3_^−^ flow battery in 0.1 M wastewater, which was operated continuously for 20 hours at 100 mA cm^−2^ and maintains the NH_4_ production rate of roughly 0.38 mmol h^−1^ cm^−2^. In addition to the remarkable stability of Ru/Co(OH)_2_ heterostructures, the lack of significant voltage degradation during the test may also be caused by the promotion of electrolyte renewal and recovery in the flow state. In addition, the promising potential of the N_2_H_4_–NO_3_^−^ flow battery has been demonstrated on electricity supply. As indicated in Fig. 13e, a low-voltage-driven anion exchange membrane hydrazine electrolyzer can be spontaneously driven by two tandem N_2_H_4_–NO_3_^−^ flow batteries, achieving a H_2_ production rate of 0.35 mmol h^−1^ cm^−2^, at output current density reaching 18.76 mA cm^−2^.

## 6. Summary and outlook

The electrocatalytic reduction of NO_*x*_^−^ to ammonia has drawn renewed interest for the restoration of the nitrogen cycle and the promotion of ammonia-based economies. This paper reviews current studies of three valorization systems based on electrocatalytic NO_*x*_^−^ conversion for energy supply and multifarious value-added chemicals synthesis, including waste treatment systems, novel electrolytic systems, and energy conversion and storage systems. In spite of the remarkable advances made in each application, some challenges and opportunities still exist.

### 6.1 Establishing uniform comparison standards and real experimental conditions

Uniform criteria are lacking in the field of NO_*x*_^−^ reduction including benchmark materials, test conditions and ammonia production units, which can potentially misguide readers and peers in judging and comparing catalyst performance. In order to achieve comparison under similar conditions, a majority of efforts focus on creating an identical test environment by establishing analogous test conditions including the electrolyte pH, NO_3_^−^/NO_2_^−^ concentration, and electrochemical test parameters such as applied potential and stability test duration. However, the pH, NO_*x*_^−^ ion concentration and operating voltage in the electrolytic environment hardly remain constant for practical application situations. Most catalysts currently developed are primarily targeted at specific pH and NO_*x*_^−^ ion concentration, ignoring the challenges of environments with variable pH, NO_*x*_^−^ ion concentration and operating voltage. Therefore, electrodes are required to be developed which generally exhibit remarkable FE and energy exchange efficiency over an extensive range of pH, NO_*x*_^−^ ion concentration and electrode potentials. In addition, the effects of wastewater constituents on reaction activity and electrode fouling still need be elucidated. For example, transition metal-based catalysts may be inactivated by complexing with interfering SCN^−^ ions in wastewater. Therefore, the effects of ionic strength, cation type, and competing anions should be thoroughly studied.

### 6.2 Deepening the mechanism research and improving the theoretical model

Although it is commonly recognized that the NO_3_^−^/NO_2_^−^ reduction catalysts in alkaline environments are more inclined to exhibit higher FE and energy conversion efficiencies compared to those in other environments, the exploration of mechanisms in different environments is unclear. Currently, apart from the knowledge of excess OH^−^ ions inhibiting the HER, more intrinsic mechanisms have not been probed accompanied by the absence of reliable experimental evidence.

Moreover, in the majority of microkinetic models currently developed for screening efficient NO_*x*_^−^ reduction catalysts, the microkinetic models are often simplified by assuming that N_ads_ is directly coupled to H_ads_. However, this theoretical model only embodies the competition from the HER, but neglects the side reaction generating N_2_ and NO. A comprehensive competitive kinetic model that can adequately display the competition of NO_*x*_^−^ reduction reactions, the HER and other side reactions generating N-containing species is currently lacking.

### 6.3 Optimizing product separation strategies

The current efforts on NO_*x*_^−^ reduction to NH_3_ are mainly focused on enhancing the economic efficiency and reducing the energy consumption of NH_3_, but the discussions on the subsequent NH_3_ separation are severely lacking. In electrochemical denitrification systems, the pH is constantly increased due to the consumption of H^+^ in the NO_*x*_^−^ reduction process. The majority of NH_3_ produced at the electrolysis interface exists in the gaseous form. In addition to the traditional techniques separating NH_3_ including the air stripping method and extraction-condensation method, acid trapping and CO_2_ capture strategies have also been demonstrated to achieve NH_3_ separation, but the availability of pure NH_3_ using such strategies is facing safety risks and cost challenges. More reasonable separation strategies need to be developed with competitive cost.

### 6.4 Constructing self-powered denitrification systems

**Fig. 14:**
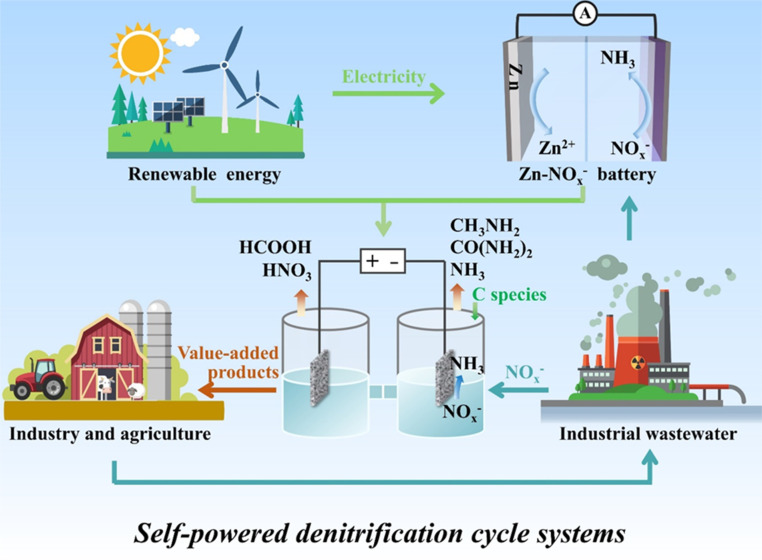
The conceptual design of a self-powered denitrification system, which employs metal–NO_*x*_^−^ batteries storing intermittent energy to supply energy for treating wastewater and producing value-added chemicals by C–N coupling reactions and alternative anodic reactions based on electrocatalytic NO_*x*_^−^ reduction.

The current electrocatalytic reduction of NO_*x*_^−^ to NH_3_ still relies on external electric power, posing a critical obstacle to practical applications in mobile devices. Therefore, a hypothetical integrated system called a “self-powered denitrification cycle system” is proposed, where NO_*x*_^−^ reduction is powered by metal–NO_*x*_^−^ batteries storing intermittent renewable energies such as wind and solar energy. This hypothetical system (Fig. 14) can be coupled with the global nitrogen cycle, where high-value-added products based on NO_*x*_^−^ reduction re-enter the nitrogen cycle to provide abundant NO_*x*_^−^ ions. The concept of self-powered denitrification cycle system not only opens up new insights for restoring the disturbed nitrogen cycle and boosting the nitrogen-based economy, but also provides a referable model for NO_*x*_^−^ reduction in a realistic scenario.

The key to the successful construction of self-powered denitrification systems lies in the development of the ammonia economy. The flourishing of the ammonia economy is linked not only to advances in catalytic technology but also to the acceptance of ammonia as an energy source by the public and the promotion of ammonia by government policies.

The chemical conversion of other N species could be introduced into the self-powered denitrification cycle system based on NO_*x*_^−^ reduction, which could further promote the restoration of the global N cycle and the prosperity of the N-based economy. N_2_, NO and NO_2_ could be converted to NO_*x*_^−^, providing a more stable approach for obtaining NO_*x*_^−^ and high-value product HNO_3_. Ammonia can be oxidized in various cells to provide hydrogen as a power source for denitrification systems.

### 6.5 From C–N coupling to artificial life synthesis

In order to enhance the efficiency and economic benefit of aqueous C–N coupling reactions and to broaden the range of N-containing organic products, it is crucial to promote the accumulation and generation of key N- and C-containing intermediates. A deeper knowledge of electrocatalytic reduction of individual organic molecules is required to genuinely facilitate the development of C–N coupling reactions.

More intriguingly, amino acid molecules have been confirmed to be formed by CO_2_ and NO_*x*_^−^. This corresponds to the first stage towards the origin of life, the evolution of inorganic molecules into organic substances in the primitive atmosphere and oceans, accompanied by energies from cosmic rays, lightning and volcanic explosion. Large accumulation of amino acid molecules could form primitive biomolecule proteins in the early oceans, and proteins could promote the process of dehydration and condensation of nucleic acids to form deoxyribonucleic acid (DNA). Subsequently, biomolecules evolved into multimolecular systems through condensation and polymerization. Finally, the organic polymolecular system evolves into primitive life, completing the intricate and lengthy process of gradual evolution from simple inorganic molecules into primitive life forms with self-replicating functions. The success of synthetic amino acids is of great importance for exploring the origin of life; moreover, with the reduction of electricity price and the development of alternative energy sources, artificial life synthesis and accelerated evolution of life is on the horizon using CO_2_, N-containing substances and H_2_O.

## Data availability

The datasets used and/or analyzed during the present study are available from the corresponding author upon reasonable request.

## Author contributions

Yi Feng: conceptualization, writing – original draft, writing – review & editing; Jin-Tao Ren: writing – review & editing; Ming-Lei Sun: writing – review & editing; Zhong-Yong Yuan: conceptualization, writing – review & editing, supervision.

## Conflicts of interest

The authors declare no conflict of interest.
